# Smart injectable hydrogels for periodontal regeneration: Recent advancements in biomaterials and biofabrication strategies

**DOI:** 10.1016/j.mtbio.2025.101855

**Published:** 2025-05-11

**Authors:** Bohan Yin, Jagan Mohan Dodda, Siu Hong Dexter Wong, G. Roshan Deen, Jeffrey S. Bate, Abhishek Pachauri, Behzad Shiroud Heidari, Tomáš Kovářík, Chi-An Luo, Shiao-Wen Tsai

**Affiliations:** a School of Medicine and Pharmacy, Ocean University of China, Qingdao, 266003, China; b Laboratory for Marine Drugs and Bioproducts, Qingdao Marine Science and Technology Center, Qingdao, 266237, China; c New Technologies – Research Centre (NTC), University of West Bohemia, Univerzitní 8, 301 00, Pilsen, Czech Republic; d Materials for Medicine Research Group, School of Medicine, The Royal College of Surgeons in Ireland (RCSI), Medical University of Bahrain, Kingdom of Bahrain; e Materials Science and Engineering, University of Utah, 122 S. Central Campus Drive, Room 304, Salt Lake City, 84112, Utah, USA; f Centre for Orthopaedic Research, School of Biomedical Science, The University of Western Australia, Nedlands, WA, 6009, Australia; g University of West Bohemia, Faculty of Mechanical Engineering, Department of Materials and Engineering Metallurgy, Univerzitní 22, 301 00, Pilsen, Czech Republic; h Department of Biomedical Engineering, Chang Gung University, Taoyuan, 33302, Taiwan; i Department of Orthopaedic Surgery, New Taipei Municipal Tucheng Hospital, New Taipei, 236, Taiwan; j Department of Periodontics, Chang Gung Memorial Hospital, Taipei, 10507, Taiwan

**Keywords:** Injectable hydrogel, Periodontal regeneration, Local drug delivery system, Tissue engineering, Periodontitis

## Abstract

Periodontitis is a globally prevalent chronic inflammatory disease that leads to periodontal pocket formation and eventually destroys tooth-supporting structures. Hence, the drastic increase in dental implants for periodontitis has become a severe clinical issue. Injectable hydrogel based on extracellular matrix (ECM) is highly biocompatible and tissue-regenerative with tailor-made mechanical properties and high payload capacity for *in situ* delivery of bioactive molecules to treat periodontitis. This therapeutic tool not only enhances the drug release efficiency and treatment efficacy but also reduces operation time. Nevertheless, it remains challenging to optimize the mechanical properties and intelligent control drug release rate of injectable hydrogels to achieve the highest therapeutic outcome. Literature precedent has shown the modulation of polymer backbones (synthetic polymers, natural polysaccharides, and proteins), crosslinking strategies, other bioactive constituents, and potentially the incorporation of nanomaterials that overall improve the desirable physiochemical and biological performances as well as biodegradability. In this review, we summarize the recent advances in the development, design, and material characterizations of common injectable hydrogels. Furthermore, we highlight cutting-edge representative examples of polysaccharide-, protein- and nanocomposite-based hydrogels that mediate regenerative factors and anti-inflammatory drugs for periodontal regeneration. Finally, we express our perspectives on potential challenges and future development of multifunctional injectable hydrogels for periodontitis.

## Graphical abstract

**Figure:**
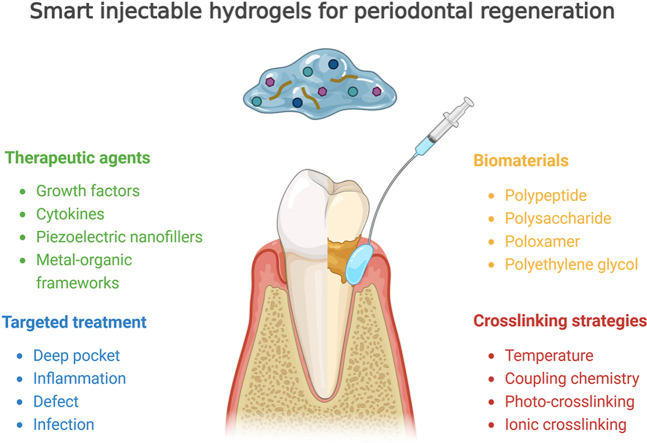

## 1 Introduction

Periodontitis is a persistent chronic inflammatory disease that causes a consequence of subgingival plaque accumulation [1]. Statistically, about 20–50 % of the global population suffers from this health issue and requires effective strategies to treat/limit the progression of this disease [2]. The symptom of the disease, including pocket formation and bone resorption, leads to alveolar bone loss in case of improper treatment. The major cause of periodontitis is bacterial activity (e.g., *Porphyromonas gingivalis*, *Tannerella forsythia* and *Treponema denticola*) that leads to pathological symptoms such as inflammatory disease [1]. In general, periodontal disease treatments are designed to suppress the inflammatory response, preventing further damage to the alveolar bone caused by periodontitis. However, most bioactive molecules have a short half-life and can be hydrolyzed or degraded by proteases within a short period. In addition, two challenges still need to be addressed to deliver drugs to periodontal pockets to treat an infection: (a) administering sufficient drugs and (b) reducing high fluid clearance rates in the pockets [3]. Bacterial infection is thought to be a factor in triggering periodontitis [4]. Therefore, inhibition of bacterial growth is crucial for the treatment of periodontitis. The existing periodontal treatments, including antibiotics application and tissue regeneration, are challenging to accomplish antibacterial effects. Conventional antibiotic treatment strategies are increasingly challenged by the development of antimicrobial resistance and the unintended disruption of the host's native microbiota. Although mechanical plaque control methods, such as routine tooth-brushing and flossing, are effective in the early stages of periodontal disease, their efficacy diminishes as the condition advances. Current therapeutic modalities, including scaling and root planning, ultrasonic instrumentation, laser-assisted debridement, and the administration of local or systemic chemotherapeutic agents are widely utilized [[5], [6], [7]]; however, they are hindered by several limitations such as technical difficulty, risk of rapid microbial recolonization, suboptimal delivery and retention of therapeutic agents within the gingival crevicular fluid, potential adverse effects, and the increasing prevalence of multidrug-resistant pathogens [8,9].

Commercially available sustained-release systems like Arestin® (minocycline microspheres) provide antibiotic release for 14–21 days, injectable hydrogels have yet to achieve optimal sequential release profiles for multiple bioactive agents needed throughout the different phases of periodontal healing. As demonstrated by Chen et al. [10], the burst release effect remains a significant challenge for hydrogel systems compared to established microsphere-based delivery methods. Further, the translation of injectable hydrogels to clinical application is hindered by manufacturing scalability issues and storage stability concerns, with Eke et al. [11] reporting that temperature-sensitive hydrogels show variable gelation kinetics under different storage conditions compared to the more stable shelf-life of conventional GTR membranes. Additionally, Larsson et al. [12] highlighted in their clinical study that while injectable hydrogels show promising results in preclinical models, randomized controlled clinical trials directly comparing their long-term efficacy against standard-of-care treatments remain limited, particularly regarding the critical clinical parameters of clinical attachment level (CAL) gain and bone fill percentage in different defect types. Also, the dynamic oral environment, such as salivary flow and tongue movement, destabilizes the retainment of injected anti-bacterial drugs to inhibit microorganisms in the defect sites. Hence, embedding biomolecules in on-demand controlled hydrogels can extend their bioavailability and prolong their efficiency period.

In addition to bactericidal treatment, restoring bone mass is another important topic in the treatment of periodontitis. One of the treatment methods for periodontal disease is to promote alveolar bone regeneration. Clinically, guided tissue regeneration (GTR) membranes are often used to induce alveolar bone regeneration, and their main function is limiting soft tissue invasion and maintaining essential space for bone regeneration [13,14]. Commercial GTR membranes fall into two categories, non-resorbable (such as extended polytetrafluoroethylene, e-PTFE) and resorbable (such as collagen membranes) [15]. The resorbable membrane does not require secondary surgery to remove and can reduce the risk of infection complications from membrane exposure. The main disadvantage is the discrepancy between the rate of membrane absorption and bone regeneration, which leads to the loss of barrier functions. To overcome the drawback, crosslinked natural or synthetic polymers are developed to prepare resorbable membranes to extend the barrier properties duration. In addition, filling the defect with acellular bone powder is an alternative treatment to GTR [15]. Bovine acellular bone powder is a commonly used material, but it still faces the problem of xeno-infection [16,17]. The incorporation of mesenchymal stem cells (derived from bone marrow, human exfoliated deciduous teeth, dental follicles, and periodontal ligaments) with biomaterial scaffolds has been studied for periodontal tissue regeneration [18,19]. Among these, periodontal ligament stem cells have demonstrated the potential for multiple differentiation with a high proliferation rate. They can differentiate into progenitor cells that form the cement, alveolar bone and ligaments. Compared to traditional methods, injectable cells/drugs containing hydrogel should be an ideal option since the narrow oral cavity space results in relatively difficult therapeutic manipulations [20].

Hydrogel biomaterials have garnered significant attention in recent years due to their unique physical, chemical, and biological properties, making them highly versatile for biomedical applications. Hydrogels are soft, semi-solid materials composed of a three-dimensional polymer network capable of retaining large amounts of water or aqueous solutions [21,22]. This water-retention capacity, combined with their biocompatibility and tunable properties, has made hydrogels ideal candidates for applications such as drug delivery, tissue engineering, and regenerative medicine. Their ability to mimic the extracellular matrix (ECM) and sustain the release of therapeutic agents further enhances their utility in these fields. Traditional hydrogels, however, face limitations in clinical applications due to their preformed nature, requiring surgical implantation, which can be inconvenient and invasive for patients [23]. To address these challenges, injectable hydrogels have emerged as a promising alternative. These hydrogels are initially in a viscous sol state, allowing them to be easily administered via syringe [24]. Upon injection, they undergo a sol-to-gel transition triggered by physiological conditions such as temperature, pH, or ionic strength, forming a stable, non-flowable gel at the target site [25]. This *in situ* gelation process enables the encapsulation and localized delivery of therapeutic agents, improving patient comfort and treatment efficacy. The injectability of these hydrogels is a key advantage, reducing the need for invasive procedures while ensuring precise delivery [26]. For their notable applications in periodontal regeneration, injectable hydrogels can be used to deliver growth factors, stem cells, or antimicrobial agents directly to periodontal defects, promoting tissue repair and regeneration while minimizing patient discomfort [27].

**Fig. 1:**
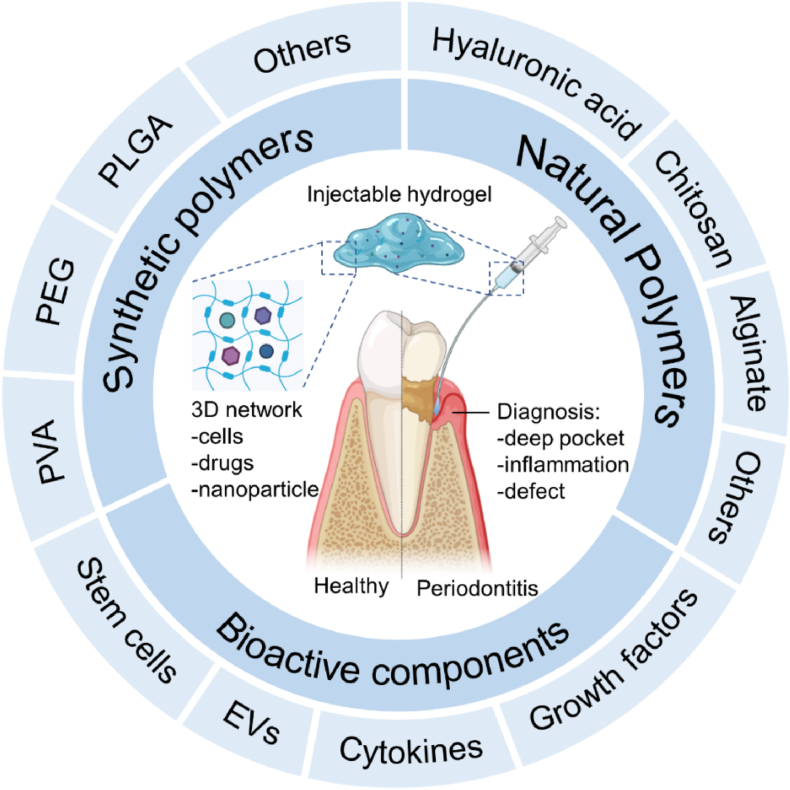
Schematic illustration of the anatomy of the periodontal defect and injectable hydrogel as a bioactive component delivery carrier for periodontal treatment. Part of the figure is created by Biorender.com.

Recently, there has been a growing interest in the use of injectable hydrogels for the treatment of dental defects owing to their unique and desirable features, including excellent tunability, *in situ* drug delivery, biocompatibility, biodegradability, stimuli responsiveness, fast recovery period, lower cost and targeted injectability [28,29]. Therefore, a periodontal defect can be effectively treated by injectable hydrogels composed of various natural, synthetic materials and/or bioactive components (Fig. 1). These hydrogels are capable of sealing the infected wound through simple injection of the pre-gel (drug-loaded) solution with a minimally invasive strategy [30,31]. Moreover, it can retain a high level of drug in the gingival crevicular fluid for the desired clinical effects on periodontal disease. The use of injectable polymeric formulations presents a significant advantage in the field of periodontal diseases and treatment due to their capacity to fill in the abscesses to aid in regeneration, recovery, and healing [3,32]. An abnormal pocket or gap (receding gum) can form between the gums and the teeth by periodontitis that increases the rate of bone destruction and affects the health of teeth [33]. Keeping good oral hygiene is the best way to prevent further periodontitis, while there is a limited strategy to regrow the lost gum. Injecting gels into the target areas as fillers to aid in directed regeneration can facilitate formulations to be introduced directly into the cavity in any form, making it a versatile delivery method. Hence, injectable hydrogel-based strategies provide a wide scope for designing carrier systems for periodontics at irregular disease sites [34]. It offers high biocompatibility with tissues and demonstrated targeted delivery with excellent amenability with patients compared to the other procedures. Despite the plethora of strategies employed to utilize injectable hydrogels for dental applications, the existing regiments have limitations concerning alveolar bone regeneration, wound healing and protection of cavities [35,36]. Many lack consistency, fluidity, and mechanical durability. Hence, developing hydrogels with *in situ* gelling, self-strengthening, and tunable properties remains critical [[37], [38], [39], [40]].

In periodontal applications, injectable hydrogels offer several clinical advantages, including direct adhesion to the infected site, enhanced therapeutic efficacy, improved patient compliance, minimal invasiveness, and ease of administration. Their high-water content, viscoelastic properties, and tissue-like properties make them particularly suitable for adapting to the complex and irregular morphology of periodontal pockets, thereby ensuring prolonged retention and localized drug delivery. These characteristics underscore the potential of injectable hydrogels as multifunctional platforms for bone regeneration and site-specific drug delivery in periodontal therapy. Several review articles have presented summaries of hydrogel-based scaffolds with highlight in promoting tissue regeneration [41], GTR membranes focusing on spatially engineered and functionally graded membranes along with in vitro antibacterial and cell-related research [13], drug delivery system by incorporating probiotics or anti-inflammatory agents [42] and composite hydrogel with improving degradation temperature [43] for periodontitis treatment and regeneration. However, no review has extensively reported the recent advances in designing injectable hydrogel for periodontal treatment and regeneration, especially from tunable mechanical properties viewpoint. This comprehensive review addresses this knowledge gap with three distinct objectives: (1) to systematically analyze recent advancements in fabrication strategies and crosslinking mechanisms that enable precise control over injectable hydrogel properties specifically engineered for the periodontal microenvironment; (2) to evaluate intelligent drug release systems tailored for periodontal pockets, including stimuli-responsive hydrogels that can respond to the pathological microenvironment of periodontitis; and (3) to assess how mechanical properties can be optimized to withstand the complex biomechanical forces in the oral cavity while maintaining injectability and *in situ* gelation capabilities. Furthermore, we analyze recent breakthroughs in smart delivery systems that enable sequential release of multiple bioactive agents (e.g., nanoceria and erythropoietin, aspirin and erythropoietin), and discuss emerging innovations in stimuli-responsive injectable hydrogels that can respond to the local microenvironment of periodontitis, providing temporally controlled therapeutic effects at the disease site.

In the later sections we reveal the suitability of injectable hydrogel for periodontics, along with other key factors that need to be considered when designing the appropriate hydrogel, the delivery method and the therapeutics delivered. Finally, we comment on recent challenges and prospects for developing injectable hydrogel for periodontics in the future. By focusing on these specific characteristics, we provide clinicians and researchers with actionable insights into how injectable hydrogels overcome limitations of conventional treatment modalities, particularly in accessing deep periodontal pockets and providing sustained therapeutic effects.

## 2 Fabrication of injectable hydrogel

### 2.1 Crosslinking strategies

The mechanical and biological properties of injectable hydrogels are highly associated with their crosslinking strategies, and even the hydrogel synthesized with the same monomeric or polymeric constituents, but different crosslinking structures can result in hydrogels of different functions. These crosslinking strategies are frequently applied to enhance the inherent physicochemical properties of the gel. On the other hand, irregular crosslinking can occur due to complex interactions between the monomer/polymer chains, crosslinking agents, or other substituents like bioactive materials, drugs, and nanomaterials. To endow injectability, hydrogels require the ability to transition from a pliable high-flow state to a stiff retentive construct when injected. Polymeric formulations used in injectable modes of delivery need to have a lower viscosity while flowing through narrow apertures can be achieved using low crosslinking densities, shear thinning, and initiated crosslinking modalities [44]. Rapid *in-situ* crosslinking of hydrogel after injection into the target site is ideal for periodontal regeneration applications, which can be achieved by chemical crosslinking agents, radiation-induced crosslinking, free radical mechanisms, enzyme-based crosslinking, and molecular crosslinking due to intermolecular interaction forces [45]. In brief, the physical properties of hydrogels can be further enhanced through the implementation of crosslinking strategies, the selection of which relies on the materials employed. Different crosslinking strategies used in injectable hydrogels are discussed in this section.

#### 2.1.1 Temperature

Hydrogels can undergo volumetric transitions such as swell, de-swell, or shrink in various osmotic gradients, or phase transitions such as sol-gel, and these changes can be integrated with stimuli-responsive functionality to allow on-demand tuning [[46], [47], [48]]. Environmental changes can not only be used to trigger responsive behavior in hydrogels but also can be interpreted as diagnostic markers for detection [49,50]. For example, thermo-sensitive hydrogel can undergo sol–gel transitions in response to temperature variation. Lower critical solution temperatures (LCST), have been widely exploited for easy and fast gelation at the physiological temperature 37°C. For instance, the LCST of poly(N-isopropylacrylamide) (PNIPAAm) is approximately 32°C, below which the polymer is hydrophilic and forms a swollen hydrogel, and above which the polymer becomes hydrophobic and the hydrogel collapses [51]. PNIPAAm's unique temperature sensitivity offers significant advantages in temperature-mediated crosslinking strategies for injectable hydrogels. With a lower critical solution temperature (LCST) range of 32–37 °C, PNIPAAm transitions from a hydrophilic sol to a hydrophobic gel upon exposure to physiological body temperature (∼37 °C). This sol-gel transformation occurs through the formation of hydrophobic interactions when the LCST is exceeded, resulting in physical crosslinking that obviates the need for chemical crosslinking agents. The physical gelation of PNIPAAm at body temperature enables minimally invasive administration, where the hydrogel remains in a liquid state during injection and subsequently solidifies *in situ* to conform to the target site. This temperature-triggered mechanism ensures high retention of the hydrogel and incorporated therapeutics, making PNIPAAm-based systems particularly well-suited for localized, controlled drug delivery in periodontal treatment. Additionally, the simplicity and effectiveness of PNIPAAm's thermal crosslinking minimize cytotoxicity risks associated with chemical crosslinkers, while maintaining good biocompatibility and tunable mechanical properties [52].

**Fig. 2:**
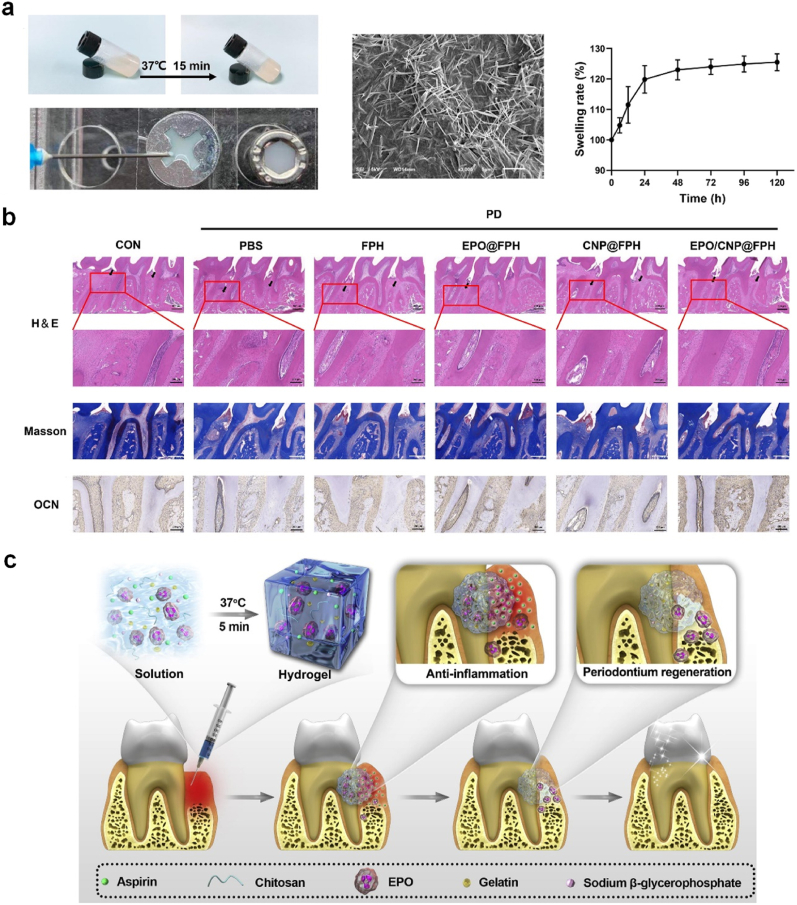
**Injectable and thermosensitive hydrogel for periodontal regeneration.** (a) Characterization of an injectable hydrogel based on Fmoc–phenylalanine bonded with diphenylalanine through π-π interaction at 37 °C to encapsulate ceria nanoparticle and erythropoietin (EPO). (b) The histological images of periodontium after treatment with hydrogels in periodontitis model *in vivo*. Images are adapted from Ref. [53], Elsevier 2023. (c) a hydrogel based on chitosan (CS), β-sodium glycerophosphate (β-GP), and gelatin to control the release of aspirin and EPO for anti-inflammation and tissue regeneration. The image is adapted from Ref. [54], Elsevier 2019.

Miao et al. reported a Fmoc-phenylalanine (Fmoc-Phe) crosslinked hydrogel via π-π interaction under the catalytic action of *Pseudomonas fluorescence lipase* for loading ceria nanoparticle (CNP) and erythropoietin (EPO) to treat periodontitis [53]. CNP is capable of regulating macrophage activation by repolarizing its phenotype from M1 to M2 by promoting reactive oxygen species (ROS) scavenging, thereby suppressing inflammation. EPO helps facilitate mineralization for vascular bone formation and osteogenic differentiation. In this study, the gelation of the pre-gel solution occurred at 37 °C for 15 min and the solution turned into an opaque white gel (Fig. 2a) [53]. The hydrogel appeared as a fibrous network structure under the observation by scanning electron microscope and showed 25.53 ± 2.77 % increased swelling rate upon incubation in phosphate buffer saline for 5 days. The hydrogel was moldable into a syringe for adapting to irregular periodontal defects. According to their findings, the injectable nanocomposite hydrogel effectively alleviated inflammation and enhanced angiopoiesis and osteogenesis of the periodontium in a rat model with satisfactory biocompatibility (Fig. 2b) [53]. In another study, Sun and colleagues employed chitosan, gelatin and β-glycerophosphate solutions to encapsulate EPO and aspirin that together formed a hydrogel within 5 min at body temperature with excellent biocompatibility (Fig. 2c) [54]. As gelatin is a type of ampholyte, it can quickly crosslink chitosan (CS) and β-glycerophosphate (β-GP) through electrostatic interaction for reduced gelation time [55]. Aspirin is a well-known non-steroidal anti-inflammatory drug to suppress inflammation and avoids the use of antibiotics. The results showed that the aspirin and EPO were sustained release over 21 days. Also, the hydrogel/aspirin/EPO group showed the effect of anti-osteoclast (prevent bone resorption) as shown by the results of tartrate-resistant acid phosphatase and receptor activator of nuclear factor-κB ligand immunostaining. Besides, this group also demonstrated significant periodontium regeneration. These studies demonstrate the importance of employing a thermo-induction strategy for *in situ* gelation at dental defect sites for effective therapy.

#### 2.1.2 Coupling chemistry

One of the key strategies for the fabrication of complex polymeric materials in hydrogels is coupling chemistry, which involves the use of chemical reactions to crosslink the hydrogel components [56]. Click reactions, a class of coupling chemistry, offer several advantages such as high yields under mild conditions, fewer by-products, high specificity, and selectivity [57]. These reactions have been widely explored for the development of injectable hydrogels, as they enable the in-situ formation of the hydrogel network, allowing for minimally invasive delivery and a high degree of control over the final material properties [56,58]. Zussman M. et al. prepared a metronidazole-eluting gelatin/alginate hydrogel as a local release anti-inflammatory drug carrier via N-(3-dimethylaminopropyl)-N-ethylcarbodiimide hydrochloride (EDC) crosslinking [59]. The sol-gel transform time was less than 10 s, so the flow of the physiological fluid did not interfere with the gelation. The hydrogel with a low alginate concentration and high gelatin concentration exhibited a short gelation time due to the entanglement in the molecular structure and an enhanced crosslinking rate. Besides, spatiotemporal self-strengthening injectable hydrogel also gained much importance for periodontal regeneration [60,61]. Yang et al. synthesized self-strengthening multifunctional hydrogels by reacting CS with aldehyde cellulose nanocrystals (DACNC) and poly(sulfobetaine methacrylate-co-glycidyl methacrylate (PSG) to produce DACNC-CS-PSG (DCP) hydrogel. Within 30 s, the amino groups of the CS and aldehyde groups of DACNC underwent a Schiff-base reaction forming an imine bond, also known as a form of dynamic crosslinking, to quickly gel the mixture. In contrast, the excess amino group of CS reacted with the epoxy group of PSG, to further crosslink the network slowly (48 h) to form the spatiotemporal self-strengthening. The tensile strength of hydrogel reached ∼210 kPa and the compressive modulus reached ∼45.6 KPa with the prolongation of reaction time, which proved the self-strengthening property of hydrogels.

#### 2.1.3 Photocrosslinking

**Fig. 3:**
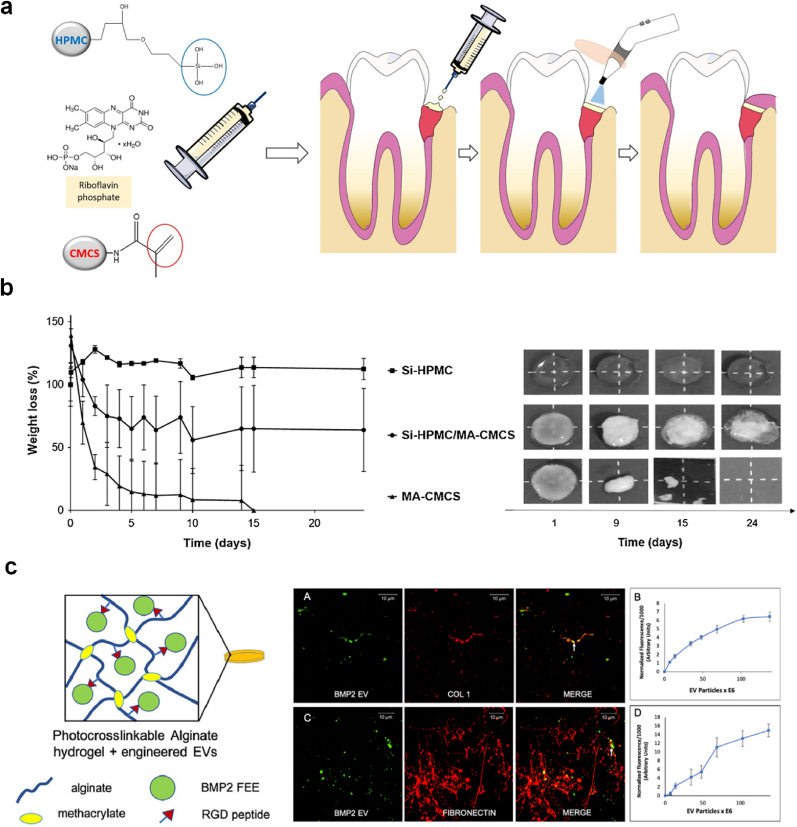
**A photocrosslinked hydrogel for dental regeneration.** (a) A liquid-to-solid *in situ* formulation of situ hydrogel membrane for guided tissue regeneration. (b) The synthesis route of methacrylated carboxymethyl chitosan and silanized hydroxypropyl methylcellulose as hydrogel prepolymers. Adapted from Ref. [64]. (c) A photocrosslinked alginate-based injectable hydrogel for prolonged release of extracellular vesicles. Adapted from reference [65].

Photocrosslinked injectable hydrogels have shown excellent efficiency in periodontal regeneration. Compared with the UV-curing system, which has been widely used to form hydrogels such as methacrylated gelatin (GelMA) [62], the visible light-curing system (such as blue light lamps in dental treatment) is safer and more readily available to prepare the hydrogels. For example, Xing et al. [63] developed CS-based hydrogel membranes by rapid photo-crosslinking with visible-light irradiation for GTR of periodontal defects. The *in-situ* gel formation occurred via photo-induced polymerization by irradiating methacrylated oxygen-substituted carboxymethyl chitosan (O-CMCS) with visible light using eosin-Y and triethanolamine as photoinitiator and 1-vinyl-2-pyrrolidinone (NVP) as a radical scavenger. The injectable CMCS-MA viscous solution turned from a liquid to gel within a quick span of 10 s when exposed to a blue light lamp (used in dentistry) in the range of 420–480 nm. The hydrogels degraded by 50 % in 28 days after subcutaneous implantation. Interestingly, the degree of substitution of the photosensitive methacrylic acid group significantly affected the physicochemical, cytotoxicity and antibacterial activity [63]. Another study also reported the use of standard dentist lamps to *in situ* photocrosslink viscous solution of methacrylated carboxymethyl chitosan (MA-CMCS) and silanized hydroxypropyl methylcellulose into an interpenetrated polymer network hydrogel membrane for GTR of periodontal defects (Fig. 3a) [64]. The degradation profiles of the hydrogels (Fig. 3b) indicate that the presence of MA-CMCS in the IPN hydrogel provides a gradual degradation mechanism, which could be beneficial for applications like guided tissue regeneration (GTR), where a controlled resorption time is necessary to match tissue healing processes. The degradation mechanism appears to be surface erosion, as evidenced by the visual changes in the hydrogels over time [64].

In a different study, photocrosslinked alginate-based injectable hydrogel was developed to encapsulate the extracellular vesicles (EVs) for prolonged delivery *in vivo* (Fig. 3c) [65]. The structural and functional integrity provided by the alginate matrix significantly improved the regeneration of bone and prolonged delivery of the therapeutics. The authors fabricated functionally engineered EVs that were able to bind mimicked peptides from collagen (DGEA, GFPGER) and fibronectin (RGD), which were decorated in alginate hydrogel. Increasing adhesive peptide amounts by varying the weight percentage of alginate increased the retention of EVs such that approximately 80 % of EVs were retained after one week in the 4 % A-RGD-FEE group. When this hydrogel was used *in vivo* in a calvarial defect model, bone regeneration was enhanced four folds compared to hydrogels without the vesicles. These results indicated the promising potential of the combined hydrogel and EVs in regenerative medicine [65]. Studies also report various crosslinking strategies for developing hydrogel matrix for periodontal regeneration, which include GelMA and methacrylic polyphosphoester (PPEMA) [66], GelMA and poly(ethylene glycol) dimethacrylate [67], GelMA and silica nanosphere [68].

Regarding the photocrosslinked hydrogels, residual photoinitiators present non-negligible toxicity concerns. These compounds generate free radicals upon light exposure to drive macromer crosslinking. While offering tunable properties through varied initiator spectra, this process poses significant biological risks. The high-energy radicals produced can induce oxidative damage to encapsulated cells, harming membranes, DNA, and proteins, ultimately compromising viability [69]. Therefore, the fundamental challenge in hydrogel photocrosslinking lies in simultaneously achieving robust material properties through sufficient crosslinking density and preserving cellular viability by reducing cytotoxic reaction products [70].

#### 2.1.4 Ionic crosslinking

The ionic crosslinking strategy has the advantage of being crosslinker-free for self-assembling polymers that can form a hydrogel network without the need for an external crosslinking agent [71]. Qu et al. developed an injectable and adhesive hydrogel system for the localized delivery of the antibiotic metronidazole (MTZ) to treat periodontitis based on incorporating CS-coated MTZ microcapsules with positively charged surfaces as an ionic crosslinker into a poly(vinyl alcohol) (PVA) with 4-carboxyphenylboronic acid (CPBA) hydrogel matrix whose backbone was negatively charged [72]. Interestingly, the hydrophobic nature of the CS coating on the microcapsules also imparted adhesive properties to the overall PVA@CS@MTZ hydrogel. The hydrogel exhibited excellent underwater adhesion to various substrates, which could enhance its retention and contact at the application site in the oral cavity. The hydrogel displayed shear-thinning behavior and self-healing properties, enabling easy injectability through a minimally invasive route. The in vitro study demonstrated that the PVA@CS@MTZ hydrogel formulation sustained the antibacterial activity against periodontal pathogens for up to 14 days. Furthermore, *in vivo* evaluation in a rat periodontitis model confirmed the desirable antibacterial efficacy of the hydrogel, which was attributed to its bioadhesive and controlled drug release characteristics [72]. The use of microcapsules as a crosslinker is a novel approach to tailor the drug release kinetics from hydrogel systems, which could have broader applications in controlled drug delivery.

For hydrogels that are prepared through ionic crosslinking strategy, their long-term stability is a critical concern in biomedical applications, owing to ion exchange and leaching [73], pH sensitivity [74], biological degradation [75]. These issues can lead to structural failure, premature degradation, or loss of functionality. To enhance stability, strategies such as dual-crosslinking (combining ionic and covalent bonds), nanocomposite reinforcement (e.g., nanoparticles or nanocellulose), polymer blending, protective surface coatings, and controlled ion release can be employed to overcome the instability. Table 1 compares the properties and applications of some hydrogels that are crosslinked via different strategies discussed above.

**Table 1:** Comparisons among hydrogels that are crosslinked via different strategies.

Crosslinking strategies	Materials	Crosslinking duration	Mechanical performance	Clinical applicability	Ref.
**Temperature**	Erythropoietin (EPO)/ceria nanoparticle (CNP) in injectable Fmoc-Phe3hydrogel (FPH)	15 min	Swelling rate increased by 25.53 ± 2.77 % after in-cubation in PBS for 5 days. Only 4.78 ± 2.93 % of the hydrogel was present after 30-day soaking	The local release of EPO/CNP@FPH modulated the immune response and macrophage polarization while enhancing osteogenic differentiation of periodontal ligament stem cells and angiogenesis in human umbilical vein endothelial cells. Results showed that CNP and EPO release reduced inflammation and promoted bone regeneration	[53]
	Chitosan (CS), b-sodium glycerophosphate (b-GP), and gelatin were used to prepare an injectable and thermosensitive hydrogel	5 min	Tensile strength of the hydrogels achieved 50.7 kPa at the strain of 76 %	Aspirin/EPO-loaded CS/β-GP/gelatin hydrogels effectively reduced inflammation and promoted periodontal regeneration in a Wistar rat periodontitis model	[54]
**Coupling chemistry**	Aldehyde cellulose nanocrystals (DACNC) and poly(sulfobetaine methacrylate-co-glycidyl methacrylate) (ploy(SBMA-co-GMA), PSG) and the other contained chitosan (CS)	30 s	Spatiotemporal adjustable mechanical strength, tensi-le strength ∼210 kPa and the compressive modulus ∼45.6 KPa with the prolo-ngation of reaction time, good swelling stability in PBS and ST solutions	Promote oral tissue regenerat-ion and bone repair	[60]
	Metronidazole-eluting gelatin and alginate crosslinked via carbodiimide	<10 s	Young's moduli range from 69 to 40 kPa and the compressive modulus decreased from 104 to 63 kPa based on gelatin and alginate concentration	The developed metronidazole-eluting hydrogels which combine controllable mechanical and physical properties together with biocompatibility and biodegradability are of high significance in the field of periodontal treatment.	[61]
**Photocrosslinking**	Methacrylated carboxymethyl chitosan	10 s	Swelling rate increased to 3000 % after incubation in PBS for 24 h	Biodegradable, perio-dontal tissue regener-ation with convenie-nce and flexibility	[63]
	Silanized hydroxypropyl methylcellulose (SiHPMC) and methacrylated carboxymethyl chitosan	120 s	Stiffness 10^3^–10^4^ Pa	High cell viability and barrier membrane effect were evaluated with soft tissue cells and ex vivo gingiva cultures.	[64]
**Ionic crosslinking**	Poly(vinyl alcohol) (PVA) crosslinked with 4-carboxyphenylboronic acid (CPBA) hydrogel matrix via metronidazole	65 s	excellent underwater adhe-sion to various substrates, shear-thinning behavior, and self-healing properties	Release behavior, an-tibacterial capacity for periodontitis trea-tment	[72]

### 2.2 Preparation strategies

The preparation of hydrogels for use in medicine and biomedical applications can be traced back to the 1960s, with the most significant application in wet and fluidic environments in the human body [[76], [77], [78], [79]]. Although these hydrogels are attractive for many biomedical applications, the kinetics of gelation during the injection process could be a limiting factor leading to the formation of heterogeneous structures at the administration site [30,80]. However, this limitation is overcome through the development of injectable dynamic hydrogels and nanocomposite hydrogels, which can reversibly transition between the sol and gel state due to their non-Newtonian properties with shear-thinning behaviors [44]. The injectability constraints are dependent on processing parameters such as needle length, geometries of syringes, rheology and volume of materials. For the application of injectable hydrogels in clinical settings, they must be compatible with a three-stage process during administration, such as (i) formulation, (ii) injection, and (iii) terminal function [30]. Several types of dynamic nanocomposite hydrogels have been developed to address the constraints related to rheology, sol-gel transitions, administration volumes, and syringe geometries [81]. When injected into tissues, these materials are initially liquid and form a physically crosslinked gel *in situ*. Moreover, these nanocomposite hydrogels can be multi-component systems containing more than one component, such as (1) polymers (natural or synthetic), and (2) nanomaterials (e.g., carbon nanotubes, graphene oxide, metallic nanoparticles, inorganic particles, and exosomes) [82]. The second component can improve its mechanical properties or increase its bioactivity.

Shaping and forming hydrogels into desired and complex 3D constructs require advanced techniques such as 3D printing and electrospinning to provide adapted structures to the defect space in patients [83,84]. Electrospinning uses the electric potential difference between a loaded syringe containing a charged polymer solution and a rotating drum to draw aligned fibers with tuned diameters dependent on nozzle gauge, viscosity, flow rate, and working distance [85]. Newer additive manufacturing methods such as extrusion, spray fusion, powder fusion, and light-based 3D printing enable a bottom-up fabrication where small amounts of droplets, layers, or powders are fused in selective geometries, and the fusion employs adhesives or initiation moieties to obtain complex constructs [86,87].

## 3 Bioactive component-based hydrogels for periodontal regeneration

Protein is assembled by units of amino acids via peptide bonds and displays unique structural and functional characteristics that are suitable for hydrogel preparation. In general, proteins are composed of three main structures: primary (linear polypeptide), secondary (α-helix and β-sheet) and tertiary (3D conformation) structures [88]. Unmodified proteins can be simply entrapped inside a polymeric matrix/hydrogel as a therapeutic being released from the network for periodontitis [89]. For example, Amelogenin (Amel), the major enamel matrix derivative (EMD), is known to boost periodontal regeneration [90]. Straumann® Emdogain® is the well-known golden standard commercial product of a synthetic gel containing porcine EMD (Amel) and propylene glycol alginate solution in conjunction with periodontal/peri-implant surgery to accelerate healing, and reduce pain and swelling [91]. Amel is highly susceptible to enzymatic degradation, which limits its effectiveness in periodontal tissue regeneration, so it requires a matrix for sustained release. Zhao et al. mixed calcium Amel with calcium alginate to prepare injectable hydrogel, and their results showed that the hybrid gel reduced wound infection, promoted healing and enhanced alveolar bone regeneration [92]. Amel can be combined with ginsenoside (Rg1), a component extracted from ginseng with an anti-inflammatory effect, to treat periodontitis and enhance periodontal regeneration [93,94]. Nevertheless, they show a short half-life and Amel can be easily degraded by proteases such as matrix metalloproteinase (MMP) *in vivo*. Thus, a locally controlled release system can resolve this limitation. Guo et al. reported a double network hydrogel formed by the Schiff based (imine) bond formation between –CHO in aldehyde-modified HA and –NH_2_ in glycol CS, as well as the dative bond between COO^−^ and Fe^3+^, as an injectable and self-healing hydrogel for controlled delivery of Rg1 and Amel to promote periodontal regeneration [28]. The degradation rate of the hydrogel was tunable based on the amount of FeCl_3_ that promoted Schiff base hydrolysis (cleavage of C=N bond) and regulated the release of the drugs via gel–sol transition. *In vivo* studies performed by injecting the gel loaded with Rg1 and Amel in the periodontitis rat model showed that the local inflammatory microenvironment was significantly suppressed by Rg1. The regeneration was successful in the bifurcation area of the root and also the first and second molars of the maxilla, indicating a promising strategy for periodontitis [28].

Except for the direct delivery of periodontal regenerative factors to the defect site, reconstruction of the hierarchical architecture of the periodontal tissues is also crucial to support a complete periodontal regeneration. In particular, the complex structure consists of the connection of soft tissues (periodontal ligament, PDL) and hard tissues (supporting structures—cementum and alveolar bone) at the periodontal defect site [95,96]. To address this issue, a multilayered/multiphasic scaffold has emerged to provide a biomimetic environment to achieve a good therapeutic outcome for customized defects of bone, cartilage and periodontal tissues [[97], [98], [99]]. Jayakumar and colleagues engineered a tri-layered nanocomposite hydrogel scaffold comprising (1) chitin–PLGA [poly(lactic-co-glycolic acid)]/nBGC (nanobioactive glass ceramic) with recombinant human cementum protein 1 (rhCEMP1) as a cementum layer, (2) chitin–PLGA with recombinant human fibroblast growth factor (rhFGF2) as a PDL layer and (3) chitin–PLGA/nBGC with platelet-rich plasma (PRP) as an alveolar bone layer for treating the periodontal defect [100]. This scaffold was highly cytocompatible and facilitated periodontal-specific differentiation in vitro: (1) cementogenic differentiation characterized by the expression of collagen type 1 (COL1) and bone sialoprotein (BSP) on the cementum layer, (2) fibrogenic differentiation analyzed through the expression of fibroblast surface protein (FSP), COL1, alkaline phosphatase (ALP) and periodontal ligament associated protein 1 (PLAP1) on the PDL layer and (3) osteogenic differentiation characterized by runt-related transcription factor 2 (RUNX2), COL1 and osteocalcin (OCN). Also, they confirmed the periodontal regenerative and defect closure ability of the scaffold *in vivo* via implanting the growth factors into rabbit maxillary periodontal defect and analyzed the results by 3D microcomputed tomography (micro-CT) reconstruction, histology and immunohistochemistry. These findings outline that multilayered nanocomposite hydrogel scaffold is a highly sound footing for periodontal defect healing and closure to reinforce tooth health. However, these hydrogels can only be pre-formed for implantation but are not injectable to facilitate irregular defect structures/wounds that cause extra steps of tailored-making the scaffold to fit the defect.

**Fig. 4:**
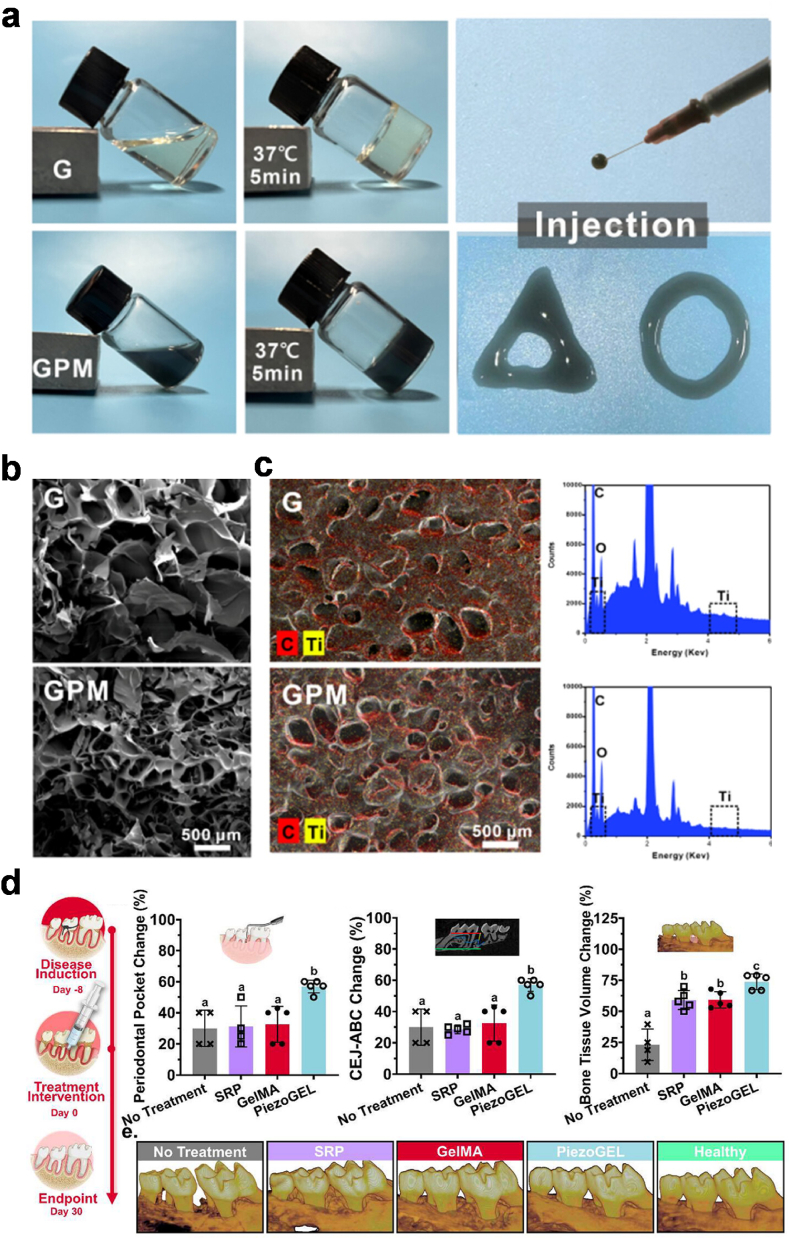
**Protein-based injectable hydrogel for periodontal regeneration.** (a) Photograph of the G/GPM hydrogels, composed of gelatin, Ti_3_C_2_T_x_ poly-L-lysine and MXene nanosheets. (b) SEM images and (c) EDS spectra of the cross-section of the injectable hydrogels. Images are adapted from Ref. [101]. Copyright 2024 American Chemical Society. (d) An injectable piezoelectric hydrogel (PiezoGEL) based on gelatin methacryloyl with biocompatible piezoelectric fillers of barium titanate that produce electrical charges when stimulated by biomechanical vibrations for enhanced osteogenesis and antibacterial effects. Images are adapted from Ref. [102]. Copyright 2023 American Chemical Society.

Yu et al. presented the development of an injectable nanocomposite hydrogel system composed of gelatin, Ti_3_C_2_T_x_ poly-L-lysine and MXene nanosheets, namely GPM, using a simple enzymatic cross-linking technique. Fig. 4a–c illustrated the gelation ability, injectability and composition of GPM composite [101]. The GPM hydrogel exhibited excellent stability, moderate tissue adhesion ability, and good mechanical behavior. The GPM hydrogel significantly inhibited the growth of *Porphyromonas gingivalis*, scavenged reactive oxygen species (ROS), attenuated inflammatory responses, and enhanced bone tissue regeneration. Importantly, the arrangement of the junctional epithelium, alveolar bone volume, and alveolar bone height in the GPM-treated periodontal disease group recovered to the levels observed in the healthy group. Hence, this study demonstrates the development of a multifunctional injectable hydrogel that shows great potential for promoting periodontal tissue regeneration and treating periodontitis through its combined antibacterial, anti-inflammatory, and bone regenerative properties [101].

Current nonsurgical treatments like scaling and root planing (SRP) combined with antibiotics have limited success and challenges like the inability to fully regenerate periodontal tissues. Roldan et al. developed an injectable piezoelectric hydrogel (PiezoGEL) by combining gelatin methacryloyl hydrogel with biocompatible piezoelectric barium titanate (BTO) fillers (Fig. 4d) [102]. They harnessed the injectability and conformability of hydrogels with the bioactive effects of piezoelectric charges. The PiezoGEL showed significant antibacterial effects, reducing pathogenic biofilm biomass (∼41 %), metabolic activity (∼75 %), and the number of viable cells (∼2–3 log) compared to hydrogels without BTO fillers in vitro. The antibacterial mechanisms were linked to reduced bacterial adhesion and increased oxidative stress. PiezoGEL also enhanced the viability and osteogenic differentiation of bone marrow stem cells, upregulating key osteogenic marker genes (RUNX2, COL1A1, and ALP) due to its electromechanical properties. In an *in vivo* mouse model, PiezoGEL effectively reduced periodontal inflammation and increased bone tissue regeneration compared to control groups in a mice model [102].

Besides, the common protein gelation can be achieved by unfolding 3D structure to a secondary structure for increased random coil content and unlimited flexibility that are accumulated into a gel matrix [103]. Together with numerous amino and carboxyl groups of proteins, the hydrophilic environment of unfolded proteins permits high swelling properties [23]. The –SH groups of cysteine in protein also assist water-holding and absorption capacity [104]. Other critical parameters, including concentration, pH and the existence of other hydrophilic functional groups, also determine the swelling ratio and mechanical properties [105]. While protein generally shows weak mechanical properties, it has been dynamically bonded with other polymeric matrices, such as polysaccharides, to support injectable, self-healing and stimuli-responsive properties for the biomedical engineering field [106,107]. Apart from crosslinking with other polymers, the modification of protein structure can also lead to gelation, such as methacrylated gelatine is often used for preparing photocrosslinking injectable hydrogels.

Previous studies have shown that the incorporation of metallic nanoparticles, such as gold, silver, silica, platinum, zinc, calcium and titanium, offers antimicrobial potential [108,109]. The formation of metal-organic frameworks (MOFs) is another promising option for antibacterial applications since they act as a reservoir of metal ions and deliver sustained release to exert bactericidal activities [[110], [111], [112]]. Liu et al. synthesized zeolitic imidazole framework-8 (ZIF) loaded gelatin methacryloyl (GelMA-ZH) that could be rapidly crosslinked under UV exposure and retained in the periodontal pocket [113]. The injected GelMA-ZH gradually released Zn^2+^ for 7 days to reduce *P. gingivalis* growth and promote alveolar bone regeneration. Their blank group showed no significant alveolar bone resorption in 7 days, while in the case of GelMA-ZH group. The alveolar bone was repaired, and the bone height was higher than those in other groups. Another example is embedding calcium peroxide in GelMA to elevate local oxygen levels, which inhibited biofilm formation since P. gingivalis only can survive in an environment with low-level oxygen [114].

Although a biomaterial scaffold may provide a good cell-laden platform to reconstruct the periodontal tissues, it lacks the complexity of the actual periodontal complex, such as PDL fibers that offer mechanical support to the teeth [41]. Ideally, PDL stem cells are a suitable tissue source candidate to promote periodontal tissue repair because of their differentiation potential of forming osteoblast-like cells, collagen-forming cells, and cementoblast-like cells [115]. Considering the limited number of PDL stem cells from mammalians or humans, induced pluripotent stem cells (iPSCs) are a powerful alternative to integrate with the scaffold as a complete biomimetic microenvironment to maximize tissue regeneration outcomes. Recently, Lo et al. reported an injectable and thermosensitive bone morphogenetic protein (BMP)-6 releasing hydrogel based on CS/gelatin/glycerol phosphate interpenetration network as a 3D environment for increasing iPSCs engraftment and survival that can increase the therapeutic potential in periodontal diseases [116]. The authors demonstrated that the hydrogel was a gel form at 4 °C and injected into the defect, where it turned into liquid at 37 °C for releasing BMP-6 and iPSCs to treat maxillary molar defect in rats. The overall therapeutic results of the experiment group (iPSC + BMP-6+hydrogel) were much better than those in other groups, including the saline group, hydrogel-only group and BMP-6+hydrogel group in terms of bone volume refraction, trabecular number, trabecular thickness and osteogenic outcomes [116]. Apart from this study, HydroMatrix is an injectable peptide nanofiber hydrogel that can self-assemble from solution precursors and respond to temperature or ionic strength for sol–gel transformation as a strategy of controlled release [117]. This product was developed for cell culture, including neural SCs, fibroblasts and keratinocytes [118,119]. Recently, HydroMatrix was shown to enhance the adhesion, survival, migration, proliferation and osteogenic differentiation of isolated primary periodontal ligament stem cells (PDLSCs), indicating a promising biocompatible scaffold for preclinical studies [120].

As periodontitis can be characterized by microbial-related and host-mediated inflammation, the polarization of macrophages plays a critical role in the pathogenesis of periodontal tissue damage [121]. In general, M1 macrophages (M1) involve in Th1-type proinflammatory immune response, while M2 macrophages (M2) involve in Th2-type anti-inflammatory immune response [122,123]. Thus, the imbalance of M1/M2 ratio can lead to alveolar bone resorption and other periodontal issues. Intriguingly, the mechanical properties of ECM, including stiffness [124], aspect ratio [125], dynamic coupling [126] and alignment [52], can guide macrophage polarization into M2 for pro-healing of bone repair and inflammation suppression in the microenvironment. Similarly, stem cells are also mechanosensitive and their osteogenic differentiation is highly influenced by the associated mechanical properties [[127], [128], [129], [130]]. Therefore, it is highly desirable to manipulate the mechanical features of dynamic hydrogel to stimulate both macrophage polarization and stem cell (e.g., PDLSC) differentiation for synergistic therapy of periodontitis. To reverse the inflammatory environment and promote tissue regeneration for periodontitis in terms of the mechanical aspect, He et al. developed a high-stiffness and injectable transglutaminase crosslinked gelatin hydrogel (TG-gel) with the encapsulation of M2-inducing immunomodulatory interleukin-4 (IL-4) and stem cell-chemoattractant stromal cell-derived factor-1α (SDF-1α) as an artificial environment for modulating immune cells, and homing the stem cells for *in situ* regeneration to repair periodontal defects [131]. Their findings demonstrated that the high-stiffness of TG-gel efficiently activated M0 (inactivated) macrophages to M1 and the presence of IL-4 further shifted them into M2 for the pro-healing environment. Besides, the inclusion of SDF-1α facilitated the stem cells to migrate toward the TG-gel, promoting the osteogenic activity of the attracted stem cells. Moreover, the co-existence of M2 and stem cells in the TG-gel further enhanced this regeneration. They confirmed these findings for in vitro 2D, and 3D cultures and the treatment of maxillary molar defects in rats. This study proves the coordinated crosstalk between stem cells and macrophages for *in situ* periodontal regeneration.

Self-assembled peptides (SAP) offer the advantages of crosslinker-free, high biocompatibility and degradability, and numerous bioactive cues [132]. Recently, a peptide termed P_11_-peptide consisting of 11 amino acids that can form an antiparallel β-sheet structure has been developed as a fibril via hydrogen bonding [133]. The nanotape can further aggregate to become a 3D fiber network that can entrap water to form an injectable and bioactive hydrogel. In particular, an 11-mer peptide P_11_–4 (Ac-QQRFEWEFEQQ-NH_2_) has been employed to treat bone defects and dental health problems [[134], [135], [136], [137]]. Mathes et al. systematically studied the effect of P_11_–4 on periodontal ligament regeneration in a 3D in vitro model by assessing the migration, ECM protein deposition and metabolic activity of human periodontal ligament fibroblasts (HPLFs) in the hydrogel [138]. Their findings showed that dentin-coated P_11_–4 promoted the secretion of ECM proteins (collagen I, III and fibrillin I of PDL), viability, and migration of HPLFs [138]. Other researchers also successfully utilized P_11_–4 to treat acute buccal bony dehiscence defects in 7 beagle dogs [139]. After 4 and 12 weeks of treatment, the defect site showed new cementum, the functionality of the newly formed periodontal ligament and recovery of height and volume of the new alveolar bone and mineral density [139]. Hence, SAP represents a new generation of biomaterials for clinical application in dentistry.

Recent advances in bioprinting have emerged for 3D tissue engineering [140]. This technology enables the manufacture of the cell-laden injectable hydrogel as a pre-defined artificial complex construct, especially useful for remodeling periodontal tissues. Among bioprinting techniques, the microextrusion method has been extensively investigated because layers of cell-laden hydrogel can be easily deposited in a computer-controlled manner and extrusion-based bioprinters are available on the market [141]. However, the number of research employing bioprinting for periodontal regeneration has been limited (up to one paper in recent years). Ivanovski et al. optimized the parameters (e.g., photoinitiator concentration, UV exposure time and power density, extrusion pressure and dispensing needle diameters) for 3D bioprinting of GelMA hydrogel that encapsulated PDL cells for the reconstruction of periodontal tissues [142]. Their findings showed maximal outcomes of cell elongation and proliferation with negligible cytotoxicity. Further optimization of the bioactivity of the printed cells and the types of biomaterials *in vivo* is required towards clinical translation. In summary, these peptide/protein properties and parameters can be optimized for treating periodontitis. Representative examples of protein-based hydrogel for periodontal regeneration have enlisted in Table 2.

**Table 2:** Representative protein-based hydrogel for periodontal regeneration.

Hydrogel components	Therapeutics release	Disease model	Experimental duration	Application and outcomes	Ref.
Polyethylene glycol (PEG) diacrylate, gingipain-responsive peptides, and dithiothreitol (DTT)	Stromal cell-derived factor-1 (SDF-1)	Rat periodontitis model via instilling *P. gingivalis* bacterial solution around the maxillary second molar	7 days (in vitro) 4 weeks (*in vivo*)	The gingipain-responsive hydrogel exhibited antibacterial functions, inhibiting the growth of *P. gingivalis* with suppressing the inflammation environment. The released SDF-1 recruited CD90^+^/CD^34−^ stromal cells for induced osteogenesis to treat periodontitis.	[143]
Tri-layered scaffold: (1) chitin–PLGA [poly(lactic-co-glycolic acid)]/nBGC (nanobioactive glass ceramic) with recombinant human cementum protein 1 (rhCEMP1), (2) chitin–PLGA with recombinant human fibroblast growth factor (rhFGF2) and (3) chitin–PLGA/nBGC with platelet-rich plasma (PRP)	rhCEMP1, rhFGF2 and PRP	Incision of tissue between incisor and molar as periodontal defect model in New Zealand white rabbits	7 and 21 days (in vitro) 1 and 3 months (*in vivo*)	The trilayered nanocomposite hydrogel scaffold promoted cementogenic, fibrogenic, and osteogenic differentiation of hDFCs on the scaffold with complete defect closure and healing with the formation of new cementum, fibrous PDL and alveolar bone with well-defined trabeculae.	[100]
HydroMatrix, self-assemble peptides	N/A	Isolated primary periodontal ligament stem cells (PDLSCs) in the hydrogel scaffold	3 weeks (in vitro)	PDLSCs showed much more mineralization in HydroMatrix culture compared to the control.	[120]
CS, gelatin and glycerol phosphate	Bone morphogenetic proteins-6 (BMP-6) and induced pluripotent stem cell (iPSCs)	Periodontal defects on the root surface of the maxillary first molar in Sprague Dawley rats	2 weeks (in vitro) 6 weeks (*in vivo*)	The injected hydrogel encapsulating iPSCs and BMP-6 in the defect promoted new bone synthesis (alkaline phosphatase- and TRAP-positive cells) and new PDL regeneration (intense Masson’ trichome staining) with reduced inflammatory level.	[116]
Gelatin and transglutaminase	SDF-1α and interleukin-4 (IL-4)	Bilateral maxillary first molars were extracted as the periodontal defect in Sprague Dawley rats	7 days (in vitro) 1, 4 and 8 weeks (*in vivo*)	The injected hydrogel suppressed the inflammation environment via shifting macrophages from M1 (proinflammatory) to M2 (prohealing) and recruit host stem cells to the defect site to orchestrate a crosstalk between stem cells and macrophages for periodontal defects.	[131]
11-mer peptide P_11_–4 (Ac-QQRFEWEFEQQ-NH_2_)	N/A	Acute buccal self-contained dehiscence-type bone defects on premolars (P1 to P4) in male Beagle dogs	4 and 12 weeks (*in vivo*)	The results demonstrated that the peptide hydrogel successfully promoted the growth of new cementum, PDL and alveolar bone (characterized by micro-computed tomography) with no adverse effect, similar to that of the group using Emdogain®.	[139]
Gelatin methacryloyl (GelMA), 2- hydroxy-4’-(2-hydroxyethoxy)-2-methylpropiophenone and lithium phenyl-2,4,6- trimethylbenzoylphosphinate (LAP)	N/A	Isolated periodontal ligament cell (PDLCs) in the bioprinted hydrogel	14 days	The PDLCs remained highly viable and proliferative in 12.5 % GelMA hydrogel and 0.05 % LAP (w/v) with extrusion pressure at 135 kPa through a 25 G dispensing needle and 20 s of UV-crosslinking.	[142]

Note: N/A denotes “not available”.

## 4 Natural polymer-based hydrogels for periodontal regeneration

Polysaccharides, as natural macromolecules, can be effectively metabolized or excreted, minimizing any prolonged accumulation or possible toxicity within a biological environment. Additionally, polysaccharides are characterized by various functional groups, such as hydroxyl, amino, and carboxyl groups, that can be chemically altered to fine-tune the characteristics of the resultant hydrogel. This section discussed several polysaccharides as material for injectable hydrogel application.

### 4.1 Chitosan-based hydrogel

The natural polymer chitosan has been widely used in injectable and injectable nanocomposite hydrogels for tissue engineering and drug delivery applications due to its excellent properties in terms of biocompatibility, cell adhesion, antimicrobial effects and biodegradability. This polymer is a linear cationic polysaccharide derived from chitin and is composed of glucosamine and *N*-acetyl glucosamine linked by β-(1,4) glycosidic bonds [144,145]. In humans, the polymer undergoes degradation by hydrolytic enzymes into chitosan (CS) oligosaccharides which are non-toxic by-products. Thermosensitive hydrogels are an ideal option for periodontal disease treatments. Injectable formulations containing CS and disodium salt of β-glycerophosphate (β-GP) form a gel around 37 °C and have been used in the encapsulation of chondrocytes in cartilage regeneration [146]. CS/β-GP hydrogels were also mixed with hydroxyapatite nanoparticles (nHA) to develop bone-related products due to their similarity with bone minerals; for instance, CS/β-GP hydrogel was mixed with hydroxyapatite nanoparticles [147]. The CS/β-GP hydrogels governed the injectability very well, hence, the addition hydroxyapatite nanoparticles did not have any negative impact on the injectability performance. The in vitro results showed that the seeded dental pulp stem cells expressed higher alkaline phosphatase activity and enhanced expression of osteogenic genes, including Runx-2, Collagen I, alkaline phosphatase, and osteocalcin in the CS/β-GP/nHA hydrogel compared to those in the CS/β-GP hydrogel [147]. Additionally, incorporating antibiotic (linezolid)-loaded degradable nanoparticles (PLGA/PLA) into CS/β-GP/hydroxyapatite nanoparticles hydrogels is effective for the long-term treatment of bone infections and the support of new tissue regeneration, while these linezolid-containing nanoparticles reduced the sol-gel gelation time by limiting the mobility of the polymer chains and facilitating the cross-linking process [148].

One of the main challenges in periodontal therapy is the regeneration of alveolar bone and the prevention of ridge reduction, and to overcome these issues, a multi-component injectable formulation consisting of CS, β-GP sodium salt, hydroxyapatite nanoparticles, and collagen (mineralized) allowed regular in vitro proliferation of mesenchymal stem cells, showing potential to treat cancerous bone defects [149]. Incorporation of the same formulation in poly(methyl methacrylate) based bone cement improved bone ingrowth and bonding [150].

Injectable bone substitutes (IBS) have drawn great attention in bone grafting as they offer effective fillings and irregular-shaped defects to reinforce the interface between dental materials and dental cement [151]. Polymethylmethacrylate (PMMA) is an ideal IBS as “bone cement”, such as cemented vertebroplasty, osteoporosis fractures and cemented tooth. However, PMMA shows inadequate stiffness and bioactivity and requires polymerization temperature [[152], [153], [154]]. To resolve these issues, Sa et al. introduced chitosan-glycerophosphate (CS-GP) into PMMA hydrogel, together with osteoconductive nano-sized hydroxyapatite (nano-HA) containing antibiotic gentamicin (GM) to form a thermosensitive hydrogel [155]. The CS-GP hydrogel acted as a pore-forming agent, creating open pores on the surface of the PMMA cement to facilitate bone tissue ingrowth and improve cement anchorage at the bone-cement interface. Additionally, the hydrogel reduced its maximum polymerization at the temperature below 30 °C, extending the working time to over 720 s, and producing cement with elasticity and a compressive yield strength ranging from 402 to 584 MPa and from 3.1 to 5.9 MPa, respectively. Incorporating nano-HA particles increased the mineralization capacity of cement without compromising its mechanical properties while incorporating GM enhanced its antibacterial activity. Importantly, the addition of nano-HA and GM-enriched CS-GP hydrogel enhanced the overall performance of PMMA cement without affecting cell survival. This hydrogel facilitated bone tissue in-growth and improved cement anchorage at the bone-cement interface in dental applications. Besides PMMA, inorganic calcium phosphates (CaP) are excellent candidates for bone regeneration as they have close chemical and crystal resemblance to bone components with good bioactivity [156]. Liao et al. constructed a porous β-tricalcium phosphate/chitosan composite scaffold to promote periodontal tissue engineering [157]. This scaffold overall enhanced the expression of bone sialoprotein and cementum attachment protein in human periodontal ligament cells (HPLCs) seeded in the composite scaffold. The expressions of other osteogenic differentiation markers such as alkaline phosphatase and osteopontin were also upregulated in the cells. Thus far, an injectable hydrogel based on CaP will be highly desirable to facilitate dental regeneration *in situ*. Fibrin is known as an insoluble protein originating from fibrinogen polymerization [158]. It can effectively support dental pulp regeneration [159]. Ducret et al. reported several fibrin–chitosan hydrogel formulations for clinical use by studying their microstructures, antimicrobial effects (anti-Enterococcus fæcalis growth), dental pulp-mesenchymal stem (DP-MSC) viability and differentiation as well as ECM deposition [160]. Their findings indicated that the formulation of 10 mg/mL fibrinogen and 0.5 % (w/w), 40 % degree of acetylation, medium molar mass chitosan showed the best anti-bacterial effect and human DP tissue neoformation. These studies manifest that chitosan is a good natural anti-bacterial hydrogel component and can be combined with various drugs and/or protein/polysaccharides for enhanced periodontal tissue engineering.

**Fig. 5:**
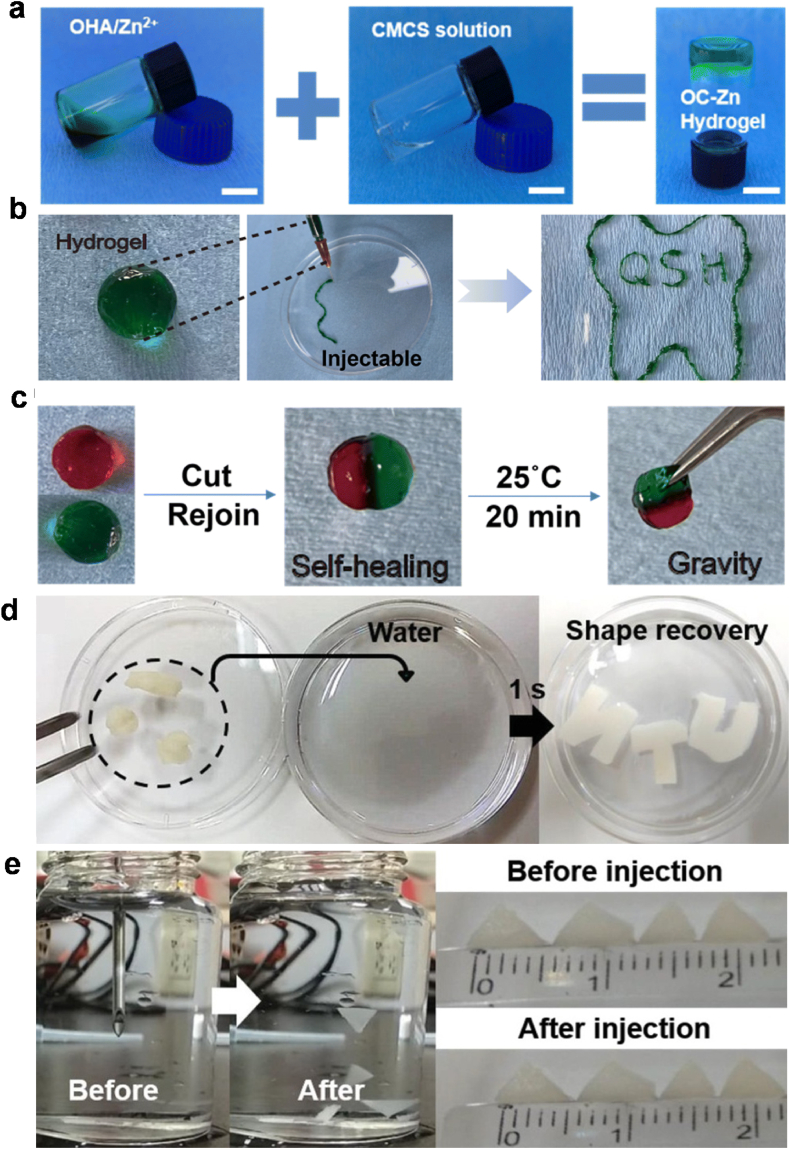
**Polysscharide-based hydrogel for periodontal regeneration.** (a) Digital photo showing the mixing of carboxymethyl chitosan (CMCS), oxidized hyaluronic acid (OHA), and zinc ions to form OC–Zn hydrogel (scale bar: 1 cm). (b) Image of the injectable of OC–Zn hydrogel into various shapes. (c) Self-healing property of OC–Zn hydrogel. Images are adapted from Ref. [161]. Copyright 2024 Royal Society of Chemistry. (d–e) Shape recovery and injectability of CS-PU cryogel (from DFPU 1.7 wt% and CS 2 wt%). (d) Cryogels distorted by external force could return to the original shape in 1 s after immersion in water. e) The cryogel (length 4 mm, thickness 1 mm) could be injected by a conventional 18-gauge needle and recover the original shape after injection in water without being distorted. Images are adapted from Ref. [162]. Copyright 2019 The Authors. Published by WILEY-VCH Verlag GmbH & Co. KGaA, Weinheim.

Yang et al. developed an injectable hydrogel that combines carboxymethyl chitosan (CMCS), oxidized hyaluronic acid (OHA), and zinc ions (Fig. 5a–c) [161]. The hydrogel formed through dynamic Schiff base bonds (dynamic crosslinking) between CMCS and OHA, as well as coordination bonds with zinc ions for self-healing properties. Their results showed that the hydrogel had good tissue adhesion, fatigue resistance, injectability, self-healing ability (Fig. 5a–c), broad-spectrum antimicrobial activity, and osteogenic activity. It showed tunable mechanical and swelling degradation with various concentrations of Zn^2+^ to form different ratios of bi-dynamic bonds. The designed OC-Zn hydrogel is proposed as a potential topical drug delivery system for the treatment of periodontitis, by providing antimicrobial and bone regenerative effects. Self-healing nanocomposite hydrogels based on glycol-CS and difunctional polyurethane (PU) have been synthesized using Schiff base reactions [162]. Depending on the properties of the Schiff bases, the hydrogels were sensitive to changes in external pH conditions and could be degraded under such environments. The self-healing type of injectable hydrogels can deliver the drug at the desired rate and enhance the recovery of periodontal hard tissues (Fig. 5d–e).

The sol-gel transition of the CS/β-GP/gelatin hydrogel occurred spontaneously when the pH of the mixed solution was adjusted to 7 and the temperature was raised to 37 °C. The gel released most of aspirin and erythropoietin in the first 8 days, and continued a sustained release till 21st day [54]. The CS/β-GP/gelatin hydrogels loaded with the drugs did not cause any toxicity and exhibited anti-inflammatory and periodontium tissue regeneration properties synergistically *in vivo* (rate model) [54]. Arpornmaeklong P. et al. demonstrated that the gelation time of CS/β-GP/collagen gel decreased significantly with increasing β-GP concentration ranging from 12.5 % to 10 % (wt/vol) [163]. The only drawback of these gels was poor mechanical strength which is normally improved through the addition of reinforcing materials. The mechanical properties of the CS-based hydrogels were significantly improved by the addition of polyethylene glycol. Acetylsalicylic acid was encapsulated in this hydrogel through electrostatic interactions [164]. The gel promoted the proliferation and osteogenic differentiation of periodontal ligament stem cells with enhanced bone regeneration. Besides, biodegradable CS-based injectable nanocomposite hydrogels containing enzymes have been used in periodontal therapy in terms of functional ligament length [165].

Chanaj-Kaczmarek et al. prepared thermosensitive hydrogels containing Scutellaria baicalensis radix lyophilized extract and CS [166]. Scutellaria baicalensis (S. baicalensis) root extract contained flavonoids like baicalin, baicalein, and wogonin, which showed anti-inflammatory and antibacterial properties. The radix lyophilized extracts with CS were added to poloxamer 407, alginate sodium, and cellulose derivatives to form thermosensitive semi-solid formulations. The presence of chitosan altered the release profile of the active compounds from the hydrogels but did not affect their in vitro permeation behavior. The authors showed synergistic effects between the S. baicalensis extract and chitosan in terms of ferrous ion-chelating activity, inhibition of hyaluronidase, and antimicrobial activity against pathogens. The thermosensitive hydrogel system showed shear-thinning properties, gelation temperature between 25 and 27 °C, and favorable mucoadhesiveness to the porcine buccal mucosa, which was enhanced by the binary mixture. The release studies demonstrated that baicalin and baicalein were released in a prolonged manner with a fast onset from the hydrogel formulations. Finally, the authors evaluated the cytoxicity of this hydrogel and demonstrated that the relative growth rate of MC3T3 cells was higher than 100 % in the presence of all cement both on the 1st day and the 3rd day, with no statistically significant difference among all groups as compared to the control. Such a formation shows a potential treatment for periodontal diseases.

For scaling and root planning for adult periodontitis, the product Periocline (Perio), which contains 2.1 % minocycline, is used in clinical practice. This commercial product is photosensitive and promotes permanent discoloration of developing teeth [167]. The adverse effects of this product could be improved using the antibiotic metronidazole. This antibiotic, however, is water-soluble and topical formulations containing metronidazole are diluted by the saliva and gingival sulcus leading to poor bioavailability. CS-based injectable hydrogels containing metronidazole microcapsules and polyvinyl alcohol were developed to overcome the dilution effect, and the hydrogel was found to be non-toxic and biocompatible [72]. The in vitro and *in vivo* experiments demonstrated good bioavailability of the loaded antibiotic making this hydrogel a promising material for periodontitis. Another example involves the use of IL-1ra, which has a short half-life, encapsulated within a CS/β-GP/gelatin hydrogel for managing periodontal inflammation [168]. In this study, IL-1ra was continuously released for up to 21 days and showed sustained inhibition of inflammation efficiency. Moreover, real-time polymerase chain reaction (PCR) showed that the expression of IL-1β, IL-6, and TNF-α inflammatory factors in cells decreased significantly after treatment with IL-1ra loaded hydrogel [168].

Hydrogels containing CS, β-GP and chitin nanowhiskers (obtained by acid hydrolysis of chitin) had enhanced mechanical properties and an exceptional gelation time of 25 s, compared to the neat hydrogel, which had a much higher gelation time of 6000 s [169]. Chitin-based injectable hydrogels incorporated with nano-fibrin and calcium sulfate (CaSO_4_), showed improved mechanical properties [170]. This chitin–CaSO_4_–nano-fibrin based injectable gel system showed improved rheology and angiogenic potential in comparison with control group (chitin only). In cell study, nano-fibrin masked the retarding effect of CaSO_4_ towards in vitro early cell attachment and angiogenesis using rabbit adipose derived mesenchymal stem cells (rASCs) and HUVECs, respectively. The spectrophotometric endpoint assay indicated rASCs osteogenesis, which was 6-fold higher in alkaline phosphatase levels and immuno-cytochemistry analysis [170]. Another interesting approach in the design of injectable nanocomposite hydrogel is to make both components (matrix and nanofiller) of the same material for tissue engineering applications [171]. CS-based hydrogels containing CS nanoparticles and loaded with tigecycline (antibiotic) and platelet-rich plasma have been developed for tissue regeneration. The injectable formulation showed shear-thinning properties and sustained release of the antibiotic against *Staphylococcus aureus* infections. A combination of CS, alginate, and fibrin forms a stiff material upon gelation due to the formation of a polyelectrolyte complex. This material promoted the adhesion of cells and allowed tissue regeneration [172].

### 4.2 HA-based hydrogels

HA is a linear polysaccharide with alternating units of β-1,4-D-glucuronic acid and β-1,3-*N*-acetyl-D-glucosamine. This natural polysaccharide is one of the important building blocks of the extracted cellular matrix of the body, and hence the molecule and its derivatives have been used in biomaterials. Although HA has excellent properties related to viscoelasticity, biocompatibility, and non-immunogenicity, it suffers from low stability that allows fast resorption in the body [173]. Many nanomaterials have been incorporated into HA hydrogels to improve their clinical applications.

HA plays an important role in the wound healing process by binding to the cell membrane receptor CD44, promoting cell proliferation and differentiation, and reducing local inflammatory processes. Therefore, large molecular mass HA-based injectable nanocomposite hydrogels have been reported to have anti-inflammatory and immunosuppressive effects. These hydrogels promote the migration of gingival fibroblast cells with beneficial effects on the inflammation caused as a result of periodontitis [174]. The mechanical properties and adhesion of HA-based hydrogels are improved by the addition of fillers. For example, methacrylic anhydride HA containing mesoporous bioactive glass (MBG) nanoparticles loaded with antibacterial drug (minocycline hydrochloride), where MBGs served as drug carriers, osteoconductive materials and enhanced the mechanical property of hydrogel. In this study, the gel inhibited the bacterial proliferation and inflammatory while promoting osteogenesis [175]. An injectable methacrylate HA-based hydrogel containing platelet lysate was developed for *in situ* release of growth factor proteins to restore the anatomy and functionality of the lost periodontal tissue [89]. The hydrogel exhibited improved rheology and the rate of degradation by the enzyme hyaluronidase was much lower. These features make this material very attractive for the treatment of periodontitis. Injectable hydrogels consisting of carbohydrazide functionalized HA and cellulose nanocrystals exhibited a slow rate of degradation. When this hydrogel was loaded with platelet lysate, it allowed the sustained release of growth factors. The hydrogel promoted the sprouting of the human dental pulp and endothelial cells [176].

Yu et al. [177] reported a hydrogel for periodontal pocket injection by incorporating copper ions (Cu^2+^) and Shed-derived exosomes (Shed-exo) within a hyaluronic acid (HA) hydrogel (Shed-Cu-HA). The Shed-Cu-HA hydrogel demonstrated enhanced viability and proliferation of human periodontal ligament stem cells with remarkable antibacterial activity against *Aggregatibacter actinomycetemcomitans* (Aa), owing to the synergistic effect of Cu^2+^ and HA. Besides, the released exosomes significantly suppressed the inflammatory response of macrophages via the IL-6/JAK2/STAT3 pathway. The synergistic osteogenic activity of Shed-exo and Cu^2+^ upregulated the expression of genes and proteins associated with osteogenic differentiation of the stem cells. In a mouse periodontitis model, the administration of Shed-Cu-HA effectively reduced the extent of inflammatory cell infiltration and bacterial infections in gingival tissues and facilitated the regeneration of periodontal bone tissues and collagen. This study showed the importance of synergistic effects on antibacterial, anti-inflammatory, and osteogenic activities for treating periodontitis [177].

**Fig. 6:**
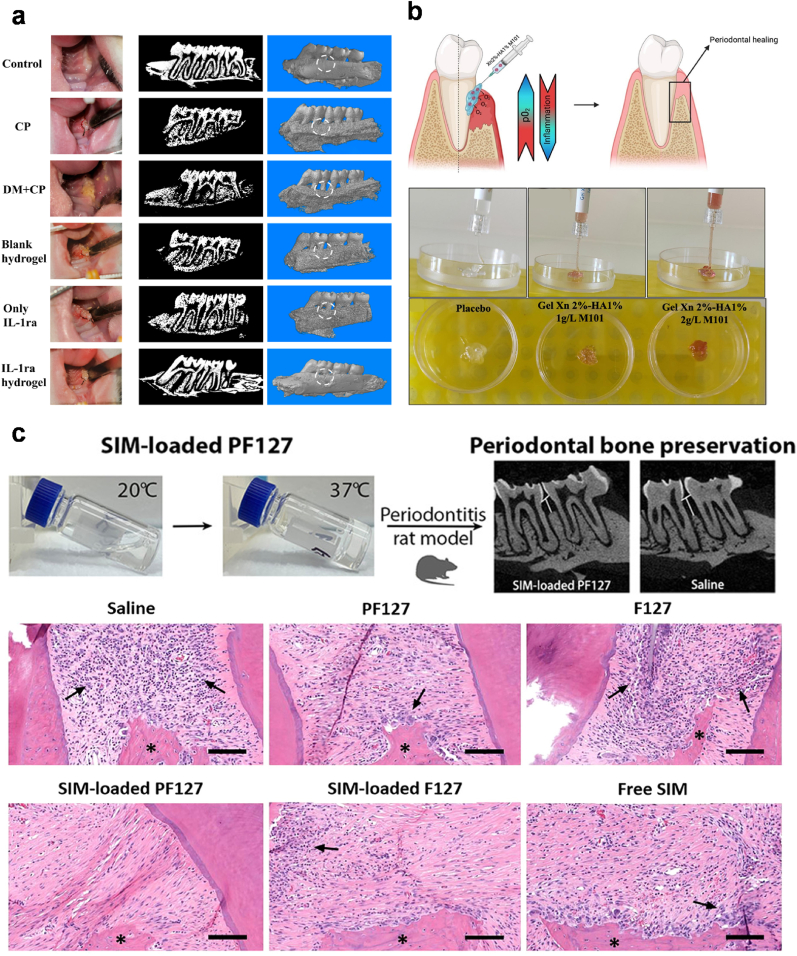
**Injectable hydrogel as local delivery carriers for periodontal disease treatment.** (a) CS/β-GP/gelatin hydrogel designed to encapsulate IL-1ra for managing periodontal inflammation. The image indicates the superior performance of CS/β-GP/gelatin formulation for sustained inhibition of inflammation over 21 days. The image is adapted from Ref. [168]. (b) A hydrogel based on 1 % hyaluronic acid (HA) and 2 % xanthan (Xn) gum containing 10 % *Arenicola marina*'s hemoglobin (M101) to inhibit the growth of *P. gingivalis*. The image is adapted from Ref. [178]. (c) Thermo-triggered hydrogel based on Pluronic® F127 conjugated with osteotropic pyrophosphate for encapsulating simvastatin and 6-bromoindirubin-3′-oxime for treating periodontitis. The image is adapted from Ref. [179].

HA can be extensively depolymerized into lower-molecular-weight polymers by hyaluronidases, β-glucuronidases, hexosaminidases, and ROS, especially when the wound is inflamed. Therefore, anti-hyaluronidase activity can enhance alveolar bone restoration. In addition, some studies have demonstrated that HA exerts bacteriostatic effects on some periodontal pathogens. Therefore, Özçelik et al. [178] embedded M101, an extract from marine worm (Arenicola marina) blood, to carry 40 times more O_2_ than human hemoglobin in an Xn/HA (xanthan gum/HA) hydrogel (Fig. 6b). Oxygen is an essential element in the wound healing process, including participating in cellular energy production, promoting angiogenesis and further enhancing cell proliferation as well as collagen synthesis. The mixed solution, which had a liquid-like shear-thinning flowing property, started flowing under applied shear stress. The media release from the gels significantly decreased the growth of P. gingivatis after 24 h. The study indicated that Xn/HA is an easily prepared carrier for M101 and has no effect on O_2_ transportation. The hydrogel showed potential for application in the recovery of periodontal lesions [178].

### 4.3 Curdlan-based hydrogels

Injectable nanocomposite hydrogels based on curdlan have also been developed for the delivery of antimicrobial therapeutics in periodontal therapy and for gene delivery [180]. Curdlan is a polysaccharide derived from bacteria and composed of β (1–3)-glucose linkages. Curdlan-based hydrogels containing polydopamine exhibited tunable properties regarding viscoelasticity, porosity and swelling behavior. The release of a model antimicrobial, acetate chlorhexidine, was studied, and the bacteriostatic rate was found to be 99.9 %. These features show the great potentials of curdlan-based hydrogels for periodontal antibacterial treatment. Other natural polymers such as chondroitin sulfate, gelatin or modified gelatin [181], zeolites and silk protein have also been evaluated for periodontal therapy [66,131,182]. Representative examples of polysaccharides-based hydrogel for periodontal regeneration have enlisted in Table 3.

**Table 3:** Representative polysaccharides-based hydrogel for periodontal regeneration.

Hydrogel major components	Other materials	Application and outcomes	Ref.
Chitosan (CS), β-glycerophosphate (β-GP), hydroxyapatite nanoparticles (nHAp)	NA	Dental pulp stem cells expressed higher alkaline phosphatase activity and osteogenic genes such as Runx-2, Collagen I, and osteocalcin	[143]
	Linezolid (antibacterial drug) in PLGA/PLA nanoparticles	PLGA/PLA nanoparticles reduced the sol-gel gelation time; long-term treatment of bone infections	[100]
	polymethylmethacrylate (PMMA), gentamicin	CS-GP hydrogel acted as a pore-forming agent, creating open pores on PMMA cement surface to facilitate bone tissue ingrowth; The polymerization temperature was reduced below 30 °C, extending the working time to over 720 s, while producing elasticity and compressive strength of the cement.	[116]
Chitosan, β-glycerophosphate, gelatine	NA	Released most of aspirin and erythropoietin in the first 8 days, and continued a sustained release till 21st day	[54]
	IL-1ra	Extension IL-1ra released for up to 21 days and showed sustained inhibition of inflammation efficiency	[168]
Chitosan, porous β-tricalcium phosphate (β-TCP)	NA	The expression of bone sialoprotein and cementum attachment protein in human periodontal ligament cells were enhanced.	[131]
Chitosan, fibrin	N/A	Anti-bacterial effect and human dental pulp tissue neoformation was observed.	[139]
Carboxymethyl chitosan (CMCS), hyaluronic acid (HA), nHAp	NA	Following mandibular incisor extraction in rats, 50 % increase in bone area; over 60 % alveolar ridge enhancement were observed after 4 weeks; wound healing completed within 7 days	[120]
Carboxymethyl chitosan (CMCS), oxidized hyaluronic acid (OHA), and zinc ions	N/A	good tissue adhesion, fatigue resistance, injectability, self-healing ability; the mechanical strength and swelling degradation of gels was depended on concentrations of Zn^2+^.	[142]
Carbohydrazide functionalized HA, cellulose nanocrystals	platelet lysate	a slow rate of degradation; promoted the sprouting of the human dental pulp and endothelial cells	[176]
Hyaluronic acid	copper ions (Cu^2+^), Shed-derived exosomes (Shed-exo)	enhanced viability and proliferation of human periodontal ligament stem cells with remarkable antibacterial activity; significantly suppressed the inflammatory response	[177]
xanthan gum/HA	an extract from marine worm blood (M101)	significantly decreased the growth of P. gingivatis after 24 h.	[178]
Curdlan	Polydopamine; acetate chlorhexidine (antimicrobial)	tunable viscoelasticity, porosity and swelling behavior; bacteriostatic rate was 99.9 %.	[180]

Note: N/A denotes “not available.

## 5 Synthetic polymer-based hydrogels

Unlike their natural counterparts, synthetic polymer-based hydrogels provide unparalleled precision in design, enabling researchers to engineer materials with exact mechanical properties, degradation rates, and bioactive cues tailored for periodontal regeneration. Free from the variability and immunogenic risks of biological sources, these hydrogels offer reproducible performance and scalability. This section explores several strategically designed synthetic hydrogels, such as PEG's bioinert versatility, PAA's mucoadhesive drug delivery, PVA's robust mechanical stability, and other innovative composites that address distinct challenges in periodontal healing.

### 5.1 Poly(ethylene glycol) (PEG)-based hydrogels

PEG hydrogels serve as effective ECM mimetics for tissue engineering due to their tunable mechanical properties, degradation profiles, and cell-binding capabilities. Their copolymerization with polylactic acid (PLA) accelerates degradation in aqueous environments. Wang et al. incorporated hydrophobic polyhedral oligomeric silsesquioxane (POSS) nanoparticles, which slowed down degradation rates while maintaining cytocompatibility, as demonstrated in PLA-PEG hydrogel systems. They developed injectable, in-situ polymerizable POSS-PEG-PLA hybrid hydrogels with controlled hydrolysis at physiological pH (7.4), gradually releasing primarily PEG and lactic acid degradation products. Importantly, this hydrogel revealed excellent biocompatibility with fibroblast cultures throughout the degradation timeline, showing no adverse effects either during initial exposure (days 1–5) or after substantial hydrogel breakdown had occurred (days 23–28), suggesting potential for minimally invasive periodontal therapies [183]. Moreover, Fraser et al. engineered PEG hydrogels with RGD/GFOGER peptides to guide periodontal regeneration by controlling periodontal ligament cell (PDLC) behavior. GFOGER-rich formulations promoted mineralization and osteogenic genes, while RGD-dominant hydrogels enhanced ALP activity via pyrophosphate regulation. This peptide-tuned hydrogel selectively direct periodontal tissue regeneration through controlled cell-matrix interactions [184]. PLA-PEG-PLA is another synthetic thermosensitive hydrogel with a transition temperature of 37 °C, which has been used for the delivery of modified mRNA [185]. The hydrogel reduced the degradation of modified mRNA and provided sustained release at the target site. It is well-known that recombinant modified mRNA encoding amelogenin (AMELX modRNA) protein has demonstrated efficacy in promoting periodontal and pulp regeneration. Based on results from a rat model, local injection of the hydrogel facilitated the sustained release of AMELX modRNA at the defect site, successfully regenerating bone and periodontal ligament tissues in periodontal defects [185].

In another study, an injectable and gingipain-responsive thermosensitive polyethylene glycol diacrylate (PEG-DA)-based hydrogel loaded with stromal cell-derived factor-1 (SDF-1) was prepared, to inhibit bacterial growth-mediated inflammation and facilitate periodontal bone regeneration [143]. SDF-1 is known as chemokine CXCL12, which can recruit endogenous periodontal ligament stem cells (PDLSCs) [186]. The short antimicrobial peptide (SAMP: CGPQRIWGQC-KKVVFKVKFK-CGPQRIWGQC) possesses strong antimicrobial function and high cytocompatibility [187]. The authors included a functional peptide module (Rgp-specific splicing site) in SAMP that was cleaved by gingipain to release SAMP for an antibacterial effect. The mechanical strength of the gel was improved by using dithiothreitol as a crosslinking agent, resulting in a complete sol-gel transformation within 10 min. They demonstrated that this hydrogel significantly decreased the proliferation of *P. gingivalis*, suppressed inflammation (Tumor Necrosis Factor-α, TNF-α and interleukin-1β, IL-1β) and promoted an early period of *in situ* periodontal tissue regeneration with pronouncedly recruited stem cells in a rat periodontitis model. Taken together, this smartly designed hydrogel promises synergistic therapy for periodontal regeneration [187].

### 5.2 Polyacrylic acid (PAA)-based hydrogels

Poly(acrylic acid) (PAA) is a hydrophilic and highly absorbent synthetic polymer containing abundant carboxyl groups, capable of forming hydrogels via either physical or chemical cross-linking methods. PAA hydrogels have been widely utilized as drug carriers due to their good bioadhesive properties and enhanced drug penetration. Qaiser et al. [188] reported a yeast–malic acid crosslinker/polyacrylic acid hydrogel as a carrier to deliver doxycycline (DX) for the treatment of periodontitis. This hydrogel exhibited porous and rough surface for DX loading, thus inducing controlled release of DX from the matrix of the hydrogel, improving patient compliance and regeneration of dental tissue [188]. In addition, a periodontitis medicine, metronidazole (MD), was loaded into PAA hydrogel using gamma-ray in a one-step process, with gel content and compressive strength of approximately 80 % and 130 kPa. This hydrogel showed no cytotoxicity and good antibacterial activity, exhibiting great potential for the treatment of periodontitis [189]. Boonlai et al. [190] developed thermosensitive poloxamer 407 (PX)/PAA hydrogels with tunable gelation temperatures (30–36 °C) for periodontal drug delivery. The hydrogel exhibited injectable shear-thinning behavior at room temperature and rapid gelation at body temperature. MD were successfully incorporated into the hydrogels with sustained release, showing promise as an injectable periodontal treatment [190].

### 5.3 Polyacrylic acid (PAA)-based hydrogels

PVA hydrogels are highly hydrophilic, mucoadhesive, and biocompatible polymers with excellent mechanical strength and chemical stability, making them ideal for biomedical and environmental applications. Glycyrrhiza glabra (GG) is well known as the bioactive component of licorice for antiviral, immunomodulatory, and antimicrobial for most oral pathogens, especially the alcoholic extracts of GG. Meanwhile, the hydroxyapatite (HAp) is the native component of teeth with enhancing bone regenerative responses, thus Chenicheri et al. [191] loaded ethanolic-crude extract of GG in alginic acid and PVA hydrogel mosaicked with HAp for periodontal regeneration application. In brief, PVA was dispersed in alginate gel by calcium ions crosslinked, forming PVA/alginate blend and dispersing HAp particles and GG extract to form the HAAPS-GG. Their result showed that HAAPS-GG inhabited the growth and survival of the major periodontal pathogens, including *S. mutans*, *L. acidophilis*, *E. faecalis* and *C. albicans* [191]. Moreover, Dong et al. [192] synthesized chitosan decorated MD microcapsules and used as a cross-linker for the preparation of a PVA injectable hydrogel by dynamic covalent bonding and ionic interaction through a 4-carboxyphenylboronic acid bridge. The release rate of the hydrophilic antibiotic from the hydrogel was slowed down by MD microcapsules. Also, the hydrophobicity of the microcapsules endows the hydrogel to be adhesive in wet conditions. Owing to its bioadhesiveness and injectable nature, the hydrogel enabled localized MD delivery to periodontal pockets, exhibiting effective antibacterial activity for 7 days *in vivo* [192].

### 5.4 Other synthetic polymer-based hydrogels

Poloxamer 407 (P407), which is also named as Pluronic® F127, is a triblock copolymer of ethylene oxide and propylene oxide with an aqueous phase at room temperature and gel form at body temperature. Thus P407-based thermoresponsive hydrogel is an attractive injectable local drug carrier due to good solubilizing capacity and low toxicity. *In vitro* evaluation of the drug release profile from P407 gel is basically based on hydrogel erosion. Therefore, numerous additives (Xanthan Gum; Carrageenan; hydroxypropyl methylcellulose, and polyvinylpyrrolidone) are incorporated P407 gel to adjust the erosion degree. Chen N. et al. synthesized a P407-PPi by conjugating pyrophosphate (PPi) to P407 to improve osteoconductivity [179]. The osteotropicity of P407-PPi can overcome the poor water solubility and no osteoconductive activity of simvastatin (SIM), which is one of the 3- hydroxy-3-methylglutaryl-cosenzyme A reductase inhibitor as an anti-inflammatory and bone anabolic agent. Their data indicated that the physical properties of P407-PPi are similar to P407, but the in vitro SIM release profiles were inferred with P407-PPi concentration. The animal histological images also showed that SIM-loaded P407-PPi hydrogel showed anti-inflammatory effects and inhibition of bone resorption when compared to the saline group (Fig. 6c). Azithromycin (AZM) is another potential drug with low water solubility for periodontitis treatments. Kerdmanee K. et al. added P407 to the pre-mix of AZM-loaded noisome and HA to develop a thermoresponsive azithromycin-loaded noisome gel (AZG) to improve the bioavailability of AZM in periodontal tissues [193]. The release profiles of the different formulations were similar, with no lag time and sustained release AZM for up to 72 h. Further analysis of the release profile found that the AZG formulation release rates followed the Korsmeyer–Peppas model, which indicated that the drug release was from polymeric matrix diffusion and erosion rather than a non-Fickian diffusion. That means the physical properties of polymer matrix played an important role in the release behaviour. The polymer matrix also facilitated the diffusion of drug due to the inhibition zone for all formulations being larger than AZM solution [193]. PLA-PEG-PLA is another synthetic thermosensitive hydrogel with a transition temperature of 37 °C, which has been used for the delivery of modified mRNA [185]. The hydrogel reduced the degradation of modified mRNA and provided sustained release at the target site. It is well-known that recombinant modified mRNA encoding amelogenin (AMELX modRNA) protein has demonstrated efficacy in promoting periodontal and pulp regeneration. Based on results from a rat model, local injection of the hydrogel facilitated the sustained release of AMELX modRNA at the defect site, successfully regenerating bone and periodontal ligament tissues in periodontal defects [185].

Dithiothreitol (DTT) can inhibit inflammation by removing ROS and preventing DNA damage by irradiation. In addition, stromal cell-derived factor-1 (SDF-1) can recruit stem cells and activate cell osteogenic ability. Liu et al. [194] combined the application of DTT and SDF-1 into PEG-DA to evaluate the synergistic effects on ROS level control and periodontal bone regeneration. In this study, the thermosensitive hydrogel was prepared by PEG-DA with DTT through Michael addition reaction and formed a gel within 5 min at 37 °C. The authors concluded that the combined application of DTT and SDF-1 hydrogel, PEGD@SDF-1, reduced the local ROS level, alleviated inflammation, and promoted *in situ* periodontal bone regeneration [194].

Glycyrrhiza glabra (GG) is well known as the bioactive component of licorice for antiviral, immunomodulatory, and antimicrobial for most oral pathogens, especially the alcoholic extracts of GG. Meanwhile, the hydroxyapatite (HAp) is the native component of teeth with enhancing bone regenerative responses, thus Chenicheri et al. [191] loaded ethanolic-crude extract of GG in alginic acid and polyvinyl alcohol (PVA) hydrogel mosaicked with HAp for periodontal regeneration application. In brief, PVA was dispersed in alginate gel by calcium ions crosslinked, forming PVA/alginate blend and dispersing HAp particles and GG extract to form the HAAPS-GG. Their result showed that HAAPS-GG inhabited the growth and survival of the major periodontal pathogens, including S. mutans, L. acidophilis, E. faecalis and C. albicans [191].

ECM in the body and around the teeth are primarily made of polysaccharides, glycans, and a variety of proteins, which are closely mimicked by natural polymers in terms of their solubility, strength, biocompatibility, and degradation [146]. Mineral-based nanoparticles such as calcium phosphate, hydroxyapatite, tricalcium phosphate, calcium sulfate, and BioGlass impart biomimetic cues that aid in guided regeneration [195]. Employing these properties into the gel composition allows for curating a microenvironment within the gel structure that is conducive to tissue growth, especially along the bone-cartilage interfaces found around the teeth. Angiogenesis, which is the formation of new blood vessels, is critical to sustaining nourishment and avoiding necrosis for GTR within periodontal implants—incorporating growth factors and drugs such as vascular endothelial growth factor, paclitaxel, and other nonsteroidal anti-inflammatory drugs into the hydrogel aids angiogenesis and tumor growth inhibition [45]. Antiseptic agents such as chlorhexidine are also explored as eluents from hydrogel fillers to mitigate infections in a periodontal pocket [196]. Composite hydrogel formulations with fibrin, collagen, HA, HAp, and phosphates enable matching the local bone and cartilage density [197,198]. Representative examples of other hydrogels for periodontal regeneration have enlisted in Table 4.

**Table 4:** Characteristics of different injectable hydrogels and their applications in periodontal treatment.

Hydrogel Type	Key Components	Physical/Chemical Properties	Crosslinking Mechanism	Periodontal Application	Advantages	Limitations	Ref.
***Natural Polymer-Based***							
**Collagen**	Type I collagen, gelatin	Biodegradable, biocompatible, moderate mechanical strength	Physical (temperature-sensitive), chemical (glutaraldehyde)	Periodontal ligament regeneration, delivery of growth factors	Promotes cell adhesion, natural ECM mimicry, hemostatic properties	Rapid degradation, batch variation, potential immunogenicity	[13,41,51]
**Chitosan**	Chitosan and its derivatives	Cationic, antimicrobial, mucoadhesive	Ionic (pH-responsive), enzymatic (genipin)	Sustained drug delivery in periodontal pockets, antibacterial agent carrier	Antimicrobial activity, bioadhesive to oral mucosa, promotes wound healing	Limited mechanical strength, variable degradation rate	[144,145,150]
**Hyaluronic Acid**	HA, modified HA derivatives	Viscoelastic, hydrophilic, biodegradable	Chemical (PEGDA), enzymatic	Anti-inflammatory agent delivery, soft tissue augmentation	Native to periodontal tissues, anti-inflammatory properties, promotes healing	Rapid enzymatic degradation, high cost	[57,62,64]
**Alginate**	Sodium alginate, calcium ions	Biocompatible, highly hydrophilic	Ionic (with divalent cations)	Delivery of antibiotics, probiotics	Ease of gelation, good biocompatibility, simple formulation	Limited mechanical properties, uncontrolled degradation	[66,79,87]
***Synthetic Polymer-Based***							
**PEG-Based**	PEG, PEGDA, PEG-peptide conjugates	Tunable mechanics, bioinert, hydrophilic	Photo-crosslinking, click chemistry	Controlled release of growth factors, cell delivery	Tunable degradation, minimal immune response, controlled release	Limited bioactivity, requires chemical modification for cell adhesion	[104,106,112]
**Pluronic**	F-127, F-68	Thermosensitive, sol-gel transition	Physical (temperature)	Local antimicrobial delivery	Thermosensitive gelation at body temperature, suitable for pocket delivery	Weak mechanical properties, rapid dissolution	[120,121]
**PNIPAAm**	PNIPAAm copolymers	Thermosensitive, LCST behavior	Physical (temperature)	Controlled drug delivery	In situ gelation at body temperature	Limited biodegradability, potential toxicity concerns	[52,129]
***Nanocomposite Hydrogels***							
**Chitosan-nHA**	Chitosan, nano-hydroxyapatite	Improved mechanical stability, osteoinductive	Ionic, physical	Alveolar bone regeneration	Enhanced mechanical properties, osteoconductivity	Complex formulation, heterogeneous degradation	[147,149]
**Collagen-Bioactive Glass**	Collagen, bioactive glass nanoparticles	Bioactive, osteoinductive, antibacterial	Physical, ionic	Periodontal bone regeneration	Mineralization promotion, sustained ion release, antibacterial	Variable mechanical properties	[51,80,82]
**Alginate-GO**	Alginate, graphene oxide	Enhanced mechanical strength, electrical conductivity	Ionic, physical	Periodontal tissue regeneration	Improved cell attachment, enhanced mechanical properties	Potential inflammatory response, limited long-term studies	[82,89]
***Stimuli-Responsive Hydrogels***							
**pH-Responsive**	PAA, PMAA derivatives	pH-dependent swelling, controlled degradation	Ionic interactions, hydrogen bonding	Targeted release in periodontal pockets	Targeted release in acidic periodontal pockets, controls bacterial acidosis	Limited mechanical stability, variable response *in vivo*	[134,136]
**Thermo-responsive**	PNIPAAm-chitosan, Pluronic-based	Sol-gel transition at body temperature	Physical (temperature), secondary interactions	In situ forming scaffolds, controlled drug release	Easy administration, *in situ* gelation	Variable gelation time, potential syneresis	[52,129,130]
**Enzyme-responsive**	MMP-cleavable peptide crosslinkers	Degradation in response to periodontal enzymes	Peptide-based crosslinks	Targeted drug release in active periodontitis	Targeted release in active inflammation zones	Complex synthesis, variable enzyme levels in patients	[138,139]

## 6 Advanced drug release mechanisms in the periodontal microenvironment

Building upon the fundamental physical characteristics, the stimulus-responsive behavior of injectable hydrogels emerges as particularly valuable in the complex oral microenvironment. Unlike static delivery systems, stimulus-responsive hydrogels modulate their physical properties and drug release kinetics in response to specific periodontal pocket conditions. In the case of pH-responsive systems, the swelling ratio and mesh size change dramatically with pH variations found in healthy (pH 6.5–7.5) versus inflamed periodontal pockets (pH 5.0–6.5) [134]. This dynamic responsiveness directly affects diffusion coefficients and release rates of incorporated therapeutics. For instance, polyacrylic acid-based hydrogels exhibit a diffusion coefficient that can vary by an order of magnitude between pH 5 and 7, creating an adaptive release system that accelerates drug delivery specifically at inflamed sites. The mechanical properties of these systems also transform with environmental triggers, with G' (storage modulus) values typically increasing 3–5 folds during pH-triggered gelation, providing enhanced retention within the pocket despite the continuous flow of gingival crevicular fluid (GCF) at rates of 20–130 μL/h depending on inflammation severity [136]. These responsive physical properties complement the baseline characteristics discussed above, allowing for dynamic adaptation to the changing periodontal environment.

In addition to these fundamental characteristics, the dual-responsive nature of chitosan-based systems offers unique advantages in periodontal applications. As a pH-responsive polymer, chitosan undergoes significant conformational changes in the periodontal pocket environment where pH fluctuates with disease activity. The protonation of amino groups (pKa ∼6.3) in acidic inflammatory environments increases polymer chain repulsion, expanding the network mesh size and accelerating drug release precisely when antimicrobial activity is most needed. Quantitative studies demonstrate that chitosan hydrogels can exhibit a 40–60 % higher release rate of antimicrobials at pH 5.5 (active periodontitis) compared to pH 7.4 (healthy tissue) [144,145].

This pH-responsive behavior creates a self-regulating delivery system that automatically adjusts release rates according to disease severity, as more acidic conditions indicate higher bacterial load and inflammation. Additionally, by incorporating temperature-responsive elements like β-glycerophosphate, chitosan-based systems can achieve dual-responsive properties, where initial temperature-triggered gelation secures the delivery system within the pocket, followed by pH-mediated release in response to local inflammation. This sequential activation mechanism is particularly valuable for maintaining therapeutic concentrations despite the challenging conditions of saliva exposure and GCF clearance in the oral cavity [150]. These responsive properties enhance the versatility of chitosan-based hydrogels beyond their inherent antimicrobial and biocompatibility characteristics described earlier.

When applied in periodontal environments, the temperature-responsive behavior of Pluronic and PNIPAAm-based hydrogels provides precise control over drug release kinetics. The mechanism involves a sol-gel transition as temperature rises above the lower critical solution temperature (LCST), typically engineered to occur between room temperature and oral cavity temperature (37 °C). This transition substantially alters the rheological properties and drug diffusion rates through the matrix.

In the complex oral environment, these thermosensitive systems demonstrate biphasic release kinetics: an initial burst release during the sol-gel transition as the network contracts, followed by sustained release governed by diffusion through the hydrogel matrix [120,121]. This profile is particularly advantageous for periodontal therapy, as it provides immediate high concentrations of antimicrobials to rapidly reduce bacterial load, followed by extended release to prevent recolonization.

The performance of thermosensitive hydrogels in the periodontal pocket is significantly influenced by local conditions. While continuous exposure to GCF can accelerate hydrogel erosion, the thermogelation process creates a structure with increased mechanical strength (typical G′ values increasing from <10 Pa in solution to >1000 Pa in gel state) that resists displacement. Furthermore, the temperature gradient that exists between the outer gingival margin and the base of deep periodontal pockets (which can differ by 1–2 °C) may create regional variations in network density, potentially resulting in spatially controlled release patterns within the same pocket [52,129]. These characteristics make Pluronic and PNIPAAm-based systems particularly suitable for the periodontal applications discussed below.

Expanding beyond conventional delivery mechanisms, stimulus-responsive hydrogels enable sophisticated drug release strategies that can be precisely tuned to periodontal pocket pathophysiology. The enzyme-responsive systems are particularly valuable in periodontal applications due to the distinct enzymatic profile of periodontitis, characterized by elevated levels of matrix metalloproteinases (MMPs, particularly MMP-8 and MMP-9), bacterial collagenases, and neutrophil elastase [138]. By incorporating enzyme-cleavable peptide sequences within the hydrogel network, these systems achieve disease-activated drug release.

The mechanism involves strategic placement of enzyme-sensitive peptide linkers that undergo specific proteolytic degradation only when target enzymes reach pathological levels. This creates an elegant feedback loop where more severe inflammation (indicated by higher enzyme levels) triggers accelerated drug release proportional to disease severity. Quantitative studies demonstrate that MMP-responsive hydrogels can increase their release rate by 200–300 % in the presence of pathological MMP concentrations (100–250 ng/ml) compared to baseline conditions [139].

Multi-responsive systems that integrate pH-, temperature-, and enzyme-responsive elements can achieve programmed sequential delivery aligned with different healing phases. For example, a dual-responsive system can employ temperature-sensitivity for initial *in situ* gelation, followed by pH or enzyme-triggered release mechanisms that respond to changing microenvironmental conditions as healing progresses. This sequential activation enables more sophisticated treatment protocols that address the shifting therapeutic needs throughout the disease-resolution-regeneration continuum of periodontal healing [134,138]. These advanced responsive mechanisms complement the conventional release systems described above, offering enhanced therapeutic potential for complex periodontal conditions.

Future studies should focus on developing adaptive systems that can respond to multiple stimuli simultaneously, providing robust release profiles even with patient-to-patient variability. Additionally, incorporating diagnostic capabilities within these hydrogels, such as colorimetric indicators that reveal local pH or enzyme activity, could enable personalized treatment monitoring, allowing clinicians to assess treatment efficacy and disease resolution non-invasively. The integration of stimulus-responsive elements with emerging technologies such as 3D printing and microfluidics may also facilitate the development of precisely engineered hydrogel geometries optimized for specific defect morphologies, further enhancing their therapeutic potential in periodontal regeneration.

## 7 Conclusions

In summary, this review discusses the use of injectable hydrogels in the treatment of dental defects. We highlight the advantages of using injectable hydrogels by their excellent tunability, *in situ* drug delivery, biocompatibility, biodegradability, stimuli responsiveness, fast recovery period, low cost, and targeted injectability. We have introduced the various materials used for the preparation of these hydrogels and the techniques employed for their fabrication, such as injection molding, extrusion, and photocuring. Although injectable hydrogels show promise for dental applications, most current formulations lack consistency and mechanical strength after injection. We suggest that synthesizing hydrogels via *in situ* gelling techniques using physical or chemical interactions can address these issues, as these techniques can modify gel properties to provide sufficient support to injured tissues and gradually strengthen themselves over time to facilitate cavity healing. We believe that our review provides valuable insights into the use of injectable hydrogels for dental applications, highlighting the challenges and opportunities for further research in this field. Developing or improving existing formulations to produce injectable hydrogels with desirable characteristics can revolutionize the treatment of dental defects.

## 8 Future direction

Due to the relatively narrow operational space and the relatively moist environment in the oral cavity, the application of injectable hydrogels in the treatment of periodontal-related diseases shows greater clinical potential compared to other material forms. The materials of hydrogels can promote bone regeneration, while the gels can encapsulate bioactive molecules to inhibit inflammation or bacterial growth and can also simultaneously embed precursor cells to accelerate tissue regeneration. However, the highly hydrated nature of hydrogels leads to relatively weak mechanical strength. Previous studies have incorporated other nanoinorganic materials to enhance strength, but this still seems insufficient to meet application demands. Therefore, although numerous different approaches have shown positive results, there has not yet been widespread clinical application.

When periodontal pockets form and alveolar bone begins to degrade, leading to periodontal ligament damage, and eventually to alveolar bone resorption and tooth loss, periodontal disease development has different stages. Regardless of modulating macrophages, reducing inflammation, preventing infection, or promoting osteogenesis, there should be different treatment requirements for different situations. Therefore, future hydrogel-designed strategies should integrate different material compositions, strengths, degradation timelines, and sufficient antibacterial drug release duration based on clinical symptoms and treatment timelines.

In the pursuit of future clinical applications of injectable hydrogels in periodontal treatment, besides focusing on the hydrogel itself, there are many other directions that can be continuously explored. For example, antibacterial efficacy can be further enhanced not only by the hydrogel itself and the drugs but also by surface modifications of the hydrogel. In addition, the hydrogel design can be combined with other treatment methods, such as low-level laser therapy, which has been proven to promote wound healing and can be designed to form crosslinked hydrogels during treatment. The establishment and selection of appropriate in vitro or animal experimental models can further accelerate clinical application.

## CRediT authorship contribution statement

**Bohan Yin:** Writing – original draft. **Jagan Mohan Dodda:** Writing – review & editing, Writing – original draft, Conceptualization. **Siu Hong Dexter Wong:** Writing – review & editing, Writing – original draft. **G. Roshan Deen:** Writing – original draft. **Jeffrey S. Bate:** Writing – original draft. **Abhishek Pachauri:** Writing – original draft. **Behzad Shiroud Heidari:** Writing – original draft. **Tomáš Kovářík:** Writing – original draft. **Chi-An Luo:** Writing – original draft. **Shiao-Wen Tsai:** Writing – review & editing, Writing – original draft.

## Declaration of competing interest

All authors declare there is no conflict of interest in this manuscript.

## Data Availability

The data described in the article are available at https://doi.org/10.5281/zenodo.10012418. We would appreciate it if other researchers could benefit from our literature and results. This will foster discussions and collaboration among scientists worldwide.

## Abbreviation list

Two-dimensional: (2D)
Three-dimensional: (3D)
Extracellular matrix: (ECM)
Guided tissue regeneration: (GTR)
Polytetrafluoroethylene: (e-PTFE)
Chitosan: (CS)
Methacrylated Gelatin: (GelMA)
Oxygen-substituted carboxymethyl chitosan: (O-CMCS)
Methacrylated carboxymethyl chitosan: (MA-CMCS)
1-vinyl-2-pyrrolidinone: (NVP)
aldehyde cellulose nanocrystals: (DACNC)
poly(sulfobetaine methacrylate-co-glycidyl methacrylate: (PSG)
DACNC-CS-PSG: (DCP)
Metal-organic frameworks: (MOFs)
Zeolitic imidazole framework-8: (ZIF)
Gelatin methacryloyl: (GelMA-Z)
Methacrylic polyphosphoester: (PPEMA)
Alveolar bone crest: (ABC)
Cementoenamel junction: (CEJ)
ZIF-8 nanoparticles: (DZIF)
Dexamethasone: (DEX)
Methacrylic polyphosphoester: (PPE)
Methacrylic polyphosphoester: (PPEMA)
Nuclear magnetic resonance spectroscopy: (NMR)
Ultraviolet–visible spectroscopy: (UV–Vis)
Fourier-transform infrared spectroscopy: (FTIR)
Electrospray ionization: (ESI)
Inductively coupled plasma: (ICP)
Matrix-assisted laser desorption/ionization: (MALDI)
Scanning electron microscopy: (SEM)
Energy dispersive x-ray: (EDX)
environmental SEM: (eSEM)
Transmission electron microscopy: (TEM)
wet scanning TEM: (wSTEM)
Cryo-scanning electron microscopy: (cryo-SEM)
Laser Confocal Scanning Microscopy: (LCSM)
Atomic force microscopy: (AFM)
Thermogravimetric analysis: (TGA)
Differential scanning calorimetry: (DSC)
Dynamic mechanical thermal analysis: (DMTA)
Enamel matrix derivative: (EMD)
Amelogenin: (Amel)
Matrix metalloproteinase: (MMP)
Polyethylene glycol diacrylate: (PEG-DA)
Periodontal ligament stem cells: (PDLSCs)
Periodontal ligament: (PDL)
recombinant human cementum protein 1: (rhCEMP1)
recombinant human fibroblast growth factor: (rhFGF2)
platelet-rich plasma: (PRP)
Bone sialoprotein: (BSP)
Collagen type 1: (COL1)
Alkaline phosphatase: (ALP)
Fibroblast surface protein: (FSP)
Periodontal ligament associated protein 1: (PLAP1)
Runt-related transcription factor 2: (RUNX2)
Osteocalcin: (OCN)
microcomputed tomography: (micro-CT)
Bone morphogenetic protein: (BMP)-6
induced pluripotent stem cells: (iPSCs)
M1 macrophages: (M1)
M2 macrophages: (M2)
Transglutaminase crosslinked gelatin hydrogel: (TG-gel)
Interleukin-4: (IL-4)
stromal cell-derived factor-1α: (SDF-1α)
Self-assembled peptides: (SAP)
Human periodontal ligament fibroblasts: (HPLFs)
β-glycerophosphate: (β-GP)
Poloxamer 407: (P407)
Chlorhexidine digluconate: (CHX)
Doxycycline hyclate: (DOXh)
Pyrophosphate: (PPi)
Simvastatin: (SIM)
Azithromycin: (AZM)
Azithromycin-loaded noisome gel: (AZG)
Dithiothreitol: (DTT)
stromal cell-derived factor-1: (SDF-1)
Quercetin: (QT)
N-(3-dimethylaminopropyl)-N-ethylcarbodiimide hydrochloride: (EDC)
Ceria nanoparticles: (CNPs)
Catalase: (CAT)
Erythropoietin: (EPO)
Fmoc-Phe3 hydrogel: (FPH)
Diphenylalanine: (Phe2)
Erythropoietin: (EPO)
Pseudomonas fluorescens lipase: (PFL)
Hyaluronic acid: (HA)
Xanthan: (Xn)
Hydroxyapatite: (HAp)
Glycyrrhiza glabra: (GG)
Polyvinyl alcohol: (PVA)
Intervertebral disc: (IVD)
Annular fibrosis: (AF)
Nucleus pulposus: (NP)
